# Facile and rapid detection of respiratory syncytial virus using metallic nanoparticles

**DOI:** 10.1186/s12951-016-0167-z

**Published:** 2016-02-27

**Authors:** Jesus Valdez, Swapnil Bawage, Idalia Gomez, Shree Ram Singh

**Affiliations:** Laboratorio de Materiales I, Facultad de Ciencias Químicas, Centro de Laboratorios Especializados, Universidad Autónoma de Nuevo León, Av. Pedro de Alba, 66451 Monterrey, Nuevo León Mexico; Center for NanoBiotechnology Research, Alabama State University, 1627, Harris way, Montgomery, AL 36104 USA

**Keywords:** RSV, Detection, Metallic nanoparticles, LSPR, SERS, Mass spectroscopy, Limit of detection, Limit of quantification, Plasmonics, Sensing

## Abstract

**Background:**

Respiratory syncytial virus (RSV) causes severe respiratory infection in infants, children and elderly. Currently, there is no effective vaccine or RSV specific drug for the treatment. However, an antiviral drug ribavirin and palivizumab is prescribed along with symptomatic treatment. RSV detection is important to ensure appropriate treatment of children. Most commonly used detection methods for RSV are DFA, ELISA and Real-time PCR which are expensive and time consuming. Newer approach of plasmonic detection techniques like localized surface plasmon resonance (LSPR) spectroscopy using metallic nanomaterials has gained interest recently. The LSPR spectroscopy is simple and easy than the current biophysical detection techniques like surface-enhanced Raman scattering (SERS) and mass-spectroscopy.

**Results:**

In this study, we utilized LSPR shifting as an RSV detection method by using an anti-RSV polyclonal antibody conjugated to metallic nanoparticles (Cu, Ag and Au). Nanoparticles were synthesized using alginate as a reducing and stabilizing agent. RSV dose and time dependent LSPR shifting was measured for all three metallic nanoparticles (non-functionalized and functionalized). Specificity of the functionalized nanoparticles for RSV was evaluated in the presence *Pseudomonas aeruginosa* and adenovirus. We found that functionalized copper nanoparticles were efficient in RSV detection. Functionalized copper and silver nanoparticles were specific for RSV, when tested in the presence of adenovirus and *P. aeruginosa*, respectively. Limit of detection and limit of quantification values reveal that functionalized copper nanoparticles are superior in comparison with silver and gold nanoparticles.

**Conclusions:**

The study demonstrates successful application of LSPR for RSV detection, and it provides an easy and inexpensive alternative method for the potential development of LSPR-based detection devices.

**Electronic supplementary material:**

The online version of this article (doi:10.1186/s12951-016-0167-z) contains supplementary material, which is available to authorized users.

## Background

Nanobiotechnology provides interdisciplinary applications such as detection, sensing, targeting, drug delivery and disease treatments [1–11]. Sensing and detection has been the subject of research in recent years using localized surface plasmon resonance (LSPR), which provides a new and easy method for their applications.

The LSPR are coherent oscillations of conducting electrons on the excited surface of a metal due to the interaction with electromagnetic radiation. These oscillations provide an extinction band in the range of infrared, visible and ultraviolet spectra. The spectral position (wavelength) of these phenomena is highly sensitive and particular to the type of metal, size, shape and the surrounding dielectric field [12] and their study is known as “plasmonics” [13–15]. The position in the spectra for the LSPR can be altered depending on the dielectric constant surrounding the metallic nanoparticles. This alteration can be observed as a blue-red shift for the LSPR peak, and it can be a useful mean for sensing applications [16].

Sensing applications involve the use of plasmon which could be enhanced and refined by improving the interactions of the electric field and metallic nanoparticles which are proportional. These improvement could help in areas of sensing because of the extraordinary sensitivity to lower concentration of chemicals [17]. Currently, the analytes studied using LSPR are metal ions [18], toxins [19], glucose [20], nucleic acids [21], molecules [22] and antigen/antibodies [23, 24]. Plasmonic nanoparticles have been used for the detection of bacteria and viruses such as *Salmonella serovars* [25] and HIV-1 [26], respectively. Detection of biological entities of respiratory diseases such as influenza viruses [3, 27] have been carried out using nanoparticle-based detection. For RSV detection, some studies reported use of surface-enhanced Raman scattering (SERS) of silver [28] nanoparticles and quantum dots (QDs-CdTe) based UV-visible spectroscopy [29, 30].

Respiratory syncytial virus (RSV) is a paramyxovirus that leads to mild, cold-like symptoms in adults and children. However, it can be more serious in infants and elderly people. Globally, RSV infection is estimated at 64 million cases and 160,000 deaths annually [31]. In the USA, the estimated infantile RSV mortality rate was shown to be more than that of influenza [32]. Therefore, early RSV detection and treatment are extremely important. It is commonly seen that RSV infection is associated with other respiratory bacterial and viral pathogens. In addition, the respiratory disease diagnosis may be difficult to differentiate between RSV and other microorganisms. The symptoms are confusing and treatment cannot be certain as the etiological agent is not known, leading to complications. For example, the respiratory infection symptoms for RSV and *Adenovirus* cannot be distinguishable during the acute phases of the illnesses [33]. RSV is responsible for promoting *Pseudomonas aeruginosa* infection [34]. In fact, mixed infection is commonly observed during respiratory illness.

The most used and commercialized method for detection of RSV is the direct fluorescence antibody (DFA) that is based on the microscopic detection with an antibody conjugated to a fluorophore. ELISA is another widely used hospital diagnostic assays for RSV detection. Real-time PCR is used to amplify and simultaneously detect or quantify a targeted DNA molecule. It is highly sensitive with very low limits of detection but it is an expensive method [35].

The biophysical methods, like PCR coupled with electrospray ionization mass spectrometry (PCR-ESI-MS) and SERS are used for RSV detection but it is largely limited for research purpose. PCR-ESI-MS is a highly sensitive and specific method even at strain level, not only for RSV but also for multiple pathogens detections [36, 37]; however, it is an expensive procedure. On the other hand, SERS is a rapid and nondestructive detection method with high sensitivity [38, 39], but the disadvantages are costs and sample preparations. However, the advantages of SERS can be availed by using LSPR spectroscopy, which serves an alternative biophysical technique to detect RSV. In this study it is showed the LSPR application of antibody-functionalized copper, silver and gold nanoparticles for the RSV detection and screened their cross-reactivity under the influence of other respiratory pathogens.

## Results

### Nanoparticles synthesis and UV-visible characterization

**Fig. 1:**
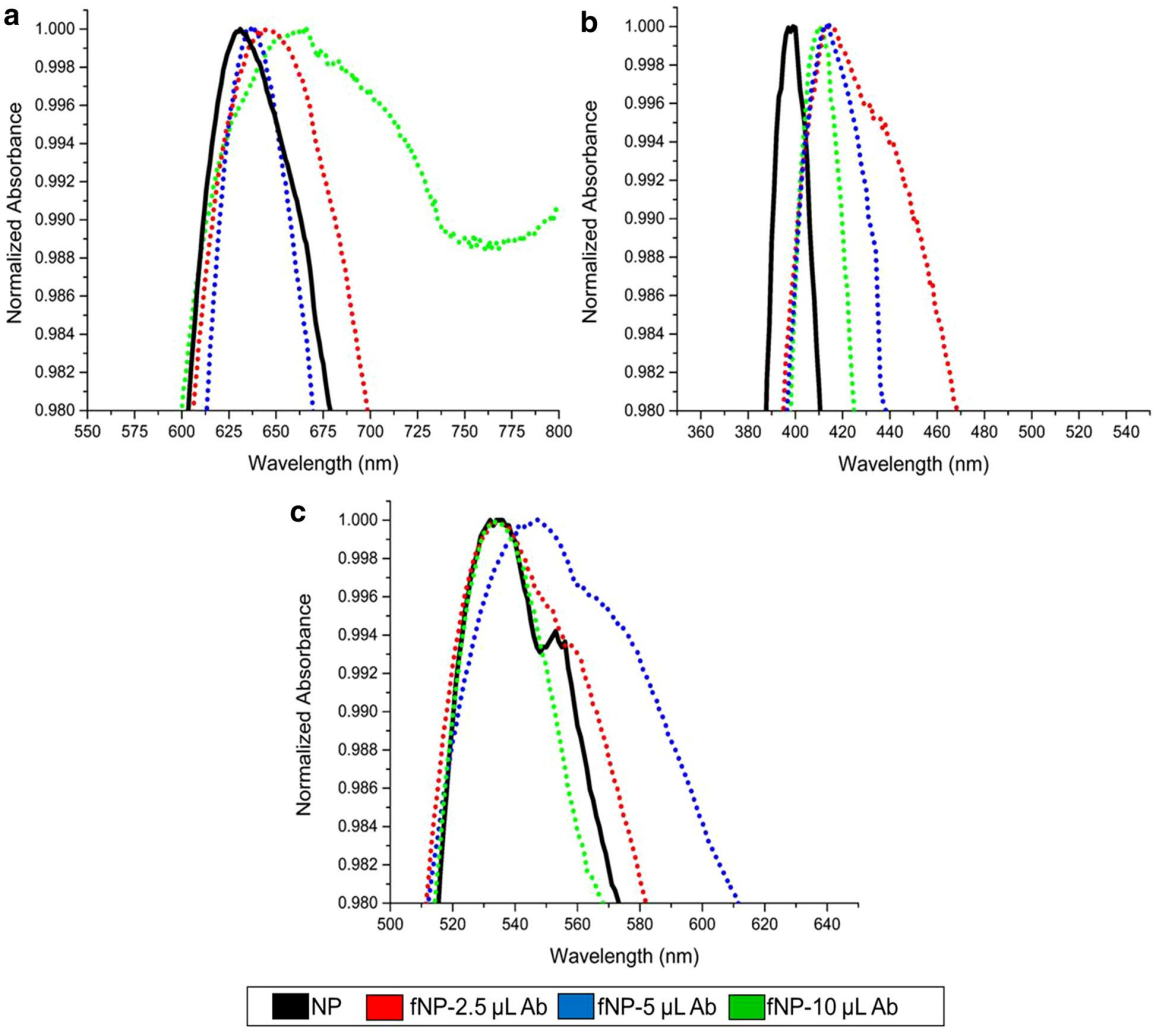
UV-visible analyses for the synthesized nanoparticles using alginate (*black line*) and their respective antibody functionalization using 2.5 (*red*), 5 (*blue*) and 10 µL (*green*) with polyclonal antibody at 3 h for copper (**a**), silver (**b**) and gold (**c**) nanoparticles

Metallic nanoparticles were synthesized by reducing and stabilizing them with alginate assisted by microware radiation. The dry weight for 200 µL of copper, silver and gold nanoparticles were 16.9 ± 0.39, 15.7 ± 0.17 and 8.3 ± 0.3 mg, respectively. The characteristic plasmonic absorption of copper, silver and gold nanoparticles was 620, 400 and 530 nm, respectively (Fig. 1).

### Functionalization of nanoparticles for optimum LSPR

All three nanoparticles were functionalized with three different volumes (2.5, 5 and 10 µL) of polyclonal antibody (4 mg/mL) for screening optimal LSPR that could be used for RSV detection. Generally, functionalization of nanoparticles reduces LSPR signal, however functionalization of nanoparticles with antibody increase the RSV detection. Therefore, fNP were appropriately selected to balance, both these desired qualities. The functionalization process was optimized at 5 µL (20 µg) of antibody for copper and gold nanoparticles and 10 µL (40 µg) for silver nanoparticles (Fig. 1). Henceforth, the nanoparticles were functionalized with these antibody concentrations for RSV detection.

### Particle size distribution and zeta potential

**Fig. 2:**
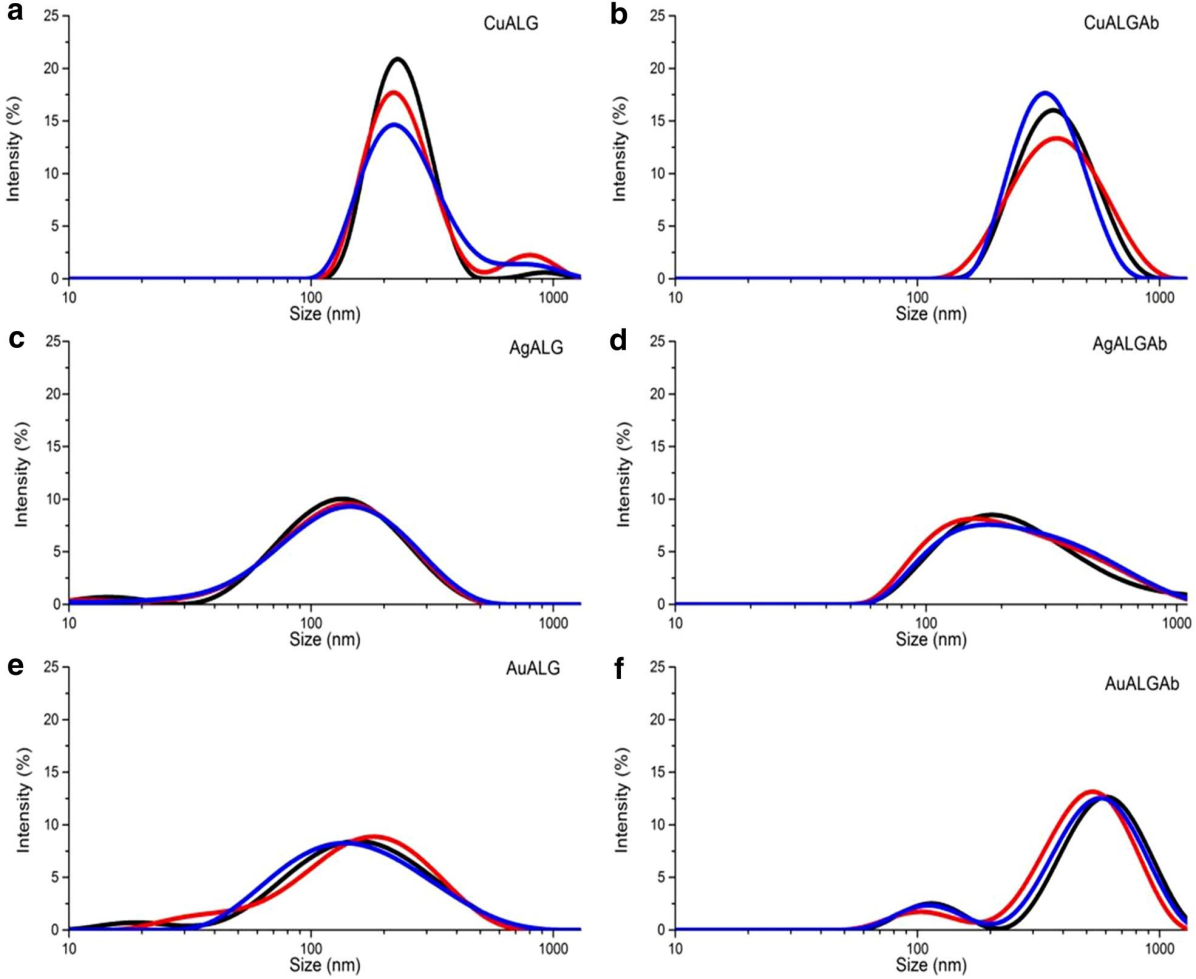
Particle size distribution for non-functionalized copper (**a**), silver (**c**) and gold (**e**) nanoparticles, and antibody functionalized (using optimized quantity) copper (**b**), silver (**d**) and gold (**f**) nanoparticles. Three separate measurements were recorded for each nanoparticle, represented with *red*, *blue* and *black* *curves*

The particle size distribution for the non-functionalized (NP) and fNP is shown in Fig. 2. Particle size distribution (Fig. 2a) for copper nanoparticles was 254 ± 11.11 nm. For silver and gold nanoparticles (Fig. 2d, e) there was a similar distribution with one peak in 10–20 nm and another one in 151 ± 0.57 and 185 ± 4.37 nm, respectively. Functionalized nanoparticles (Fig. 2b, e, g) were larger in size than NP nanoparticles. For copper and silver nanoparticles, sizes increased up to130 nm approximately, and for gold nanoparticles, two peaks of 118 ± 2.82 and 585 ± 52 nm were observed.

**Fig. 3:**
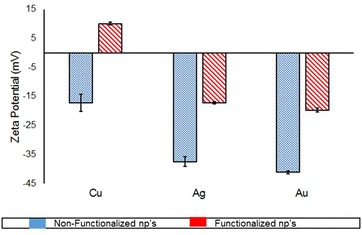
Zeta potential for non-functionalized (*blue*) and antibody functionalized copper, silver and gold nanoparticles (*red*) dispensed in water at pH of 6.58 measured at room temperature

The zeta potential for copper nanoparticles was −17.2 mV, and upon functionalization the potential changed to 10 mV. Silver and gold nanoparticles showed a potential of −37 and −40 mV respectively, and after functionalization they had potentials of −18 mV for silver and −20 mV for gold (Fig. 3).

### Determination of antibody functionalized on the nanoparticles

The supernatant and washes from the antibody fNP were subjected to protein estimation using the BCA (Thermo-Scientific, NY, USA). The antibody attached to the surface of nanoparticles was 18.56 ± 0.38 µg of 20 µg (93.3 %) for copper, 33. 60 ± 0.49 µg of 40 µg (84 %) for silver and 11.04 ± 1.6 µg of 20 µg (55.2 %) for gold.

### Field emission-scanning electron microscopy

**Fig. 4:**
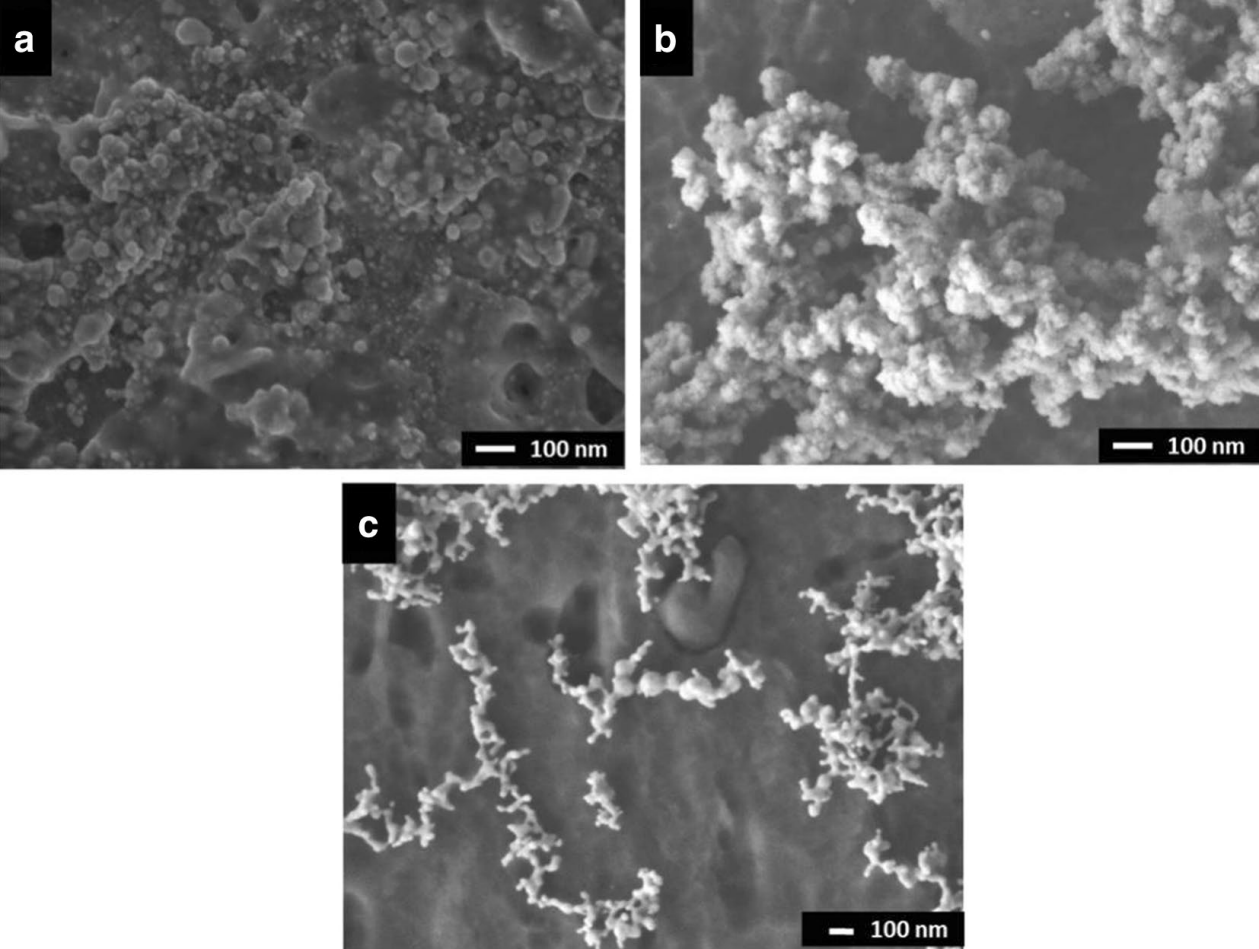
Field-emission scanning electron micrographs of non-functionalized copper (**a**), silver (**b**) and gold (**c**) nanoparticles

FE-SEM micrographs for NP indicate the size of all NPs to be less than 100 nm with few visible agglomerations (Fig. 4).

### RSV detection

**Fig. 5:**
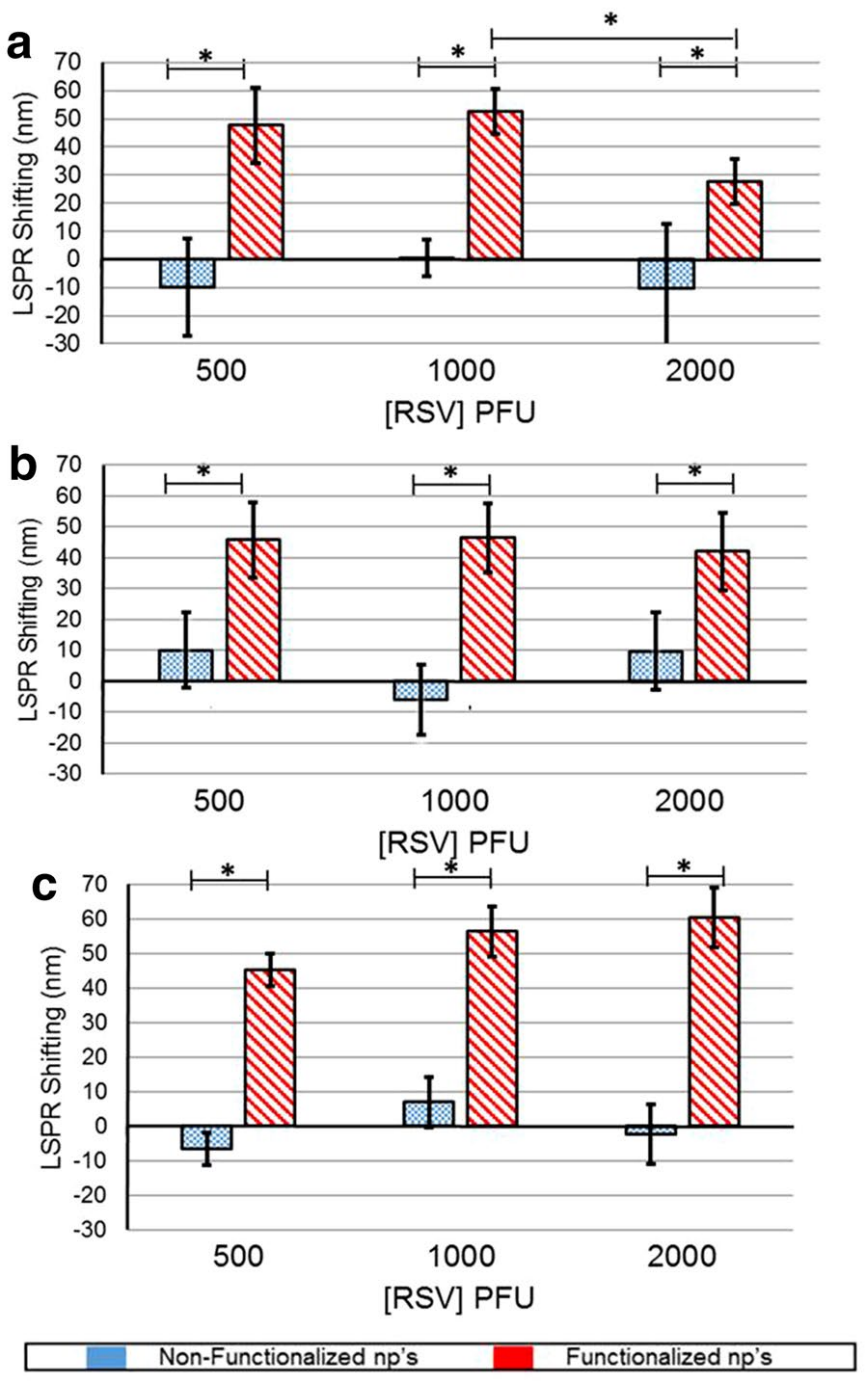
*Graph* illustrates the LSPR shifting at different titres of RSV at 30 min (**a**), 60 min (**b**) and 120 min (**c**) for antibody-functionalized (*red*) and non-functionalized (*blue*) copper nanoparticles. The *asterisk* symbol represents the significance p < 0.05

The magnitude of interaction between the RSV and functionalized nanoparticle is reflected as a corresponding measurable LSPR shift. The LSPR shifting for NPs and fNPs in presence of RSV is compared with the LSPR of NPs (see Additional file 1). There was shifting of LSPR with all RSV titer for the functionalized copper nanoparticles at 30, 60 and 120 min (Fig. 5). Increasing the time of contact between nanoparticles and RSV (all dose) did not have significant difference, except at 30 min time point for 2000 PFU RSV. The non-functionalized copper nanoparticles did not show significant change in the shifting.

**Fig. 6:**
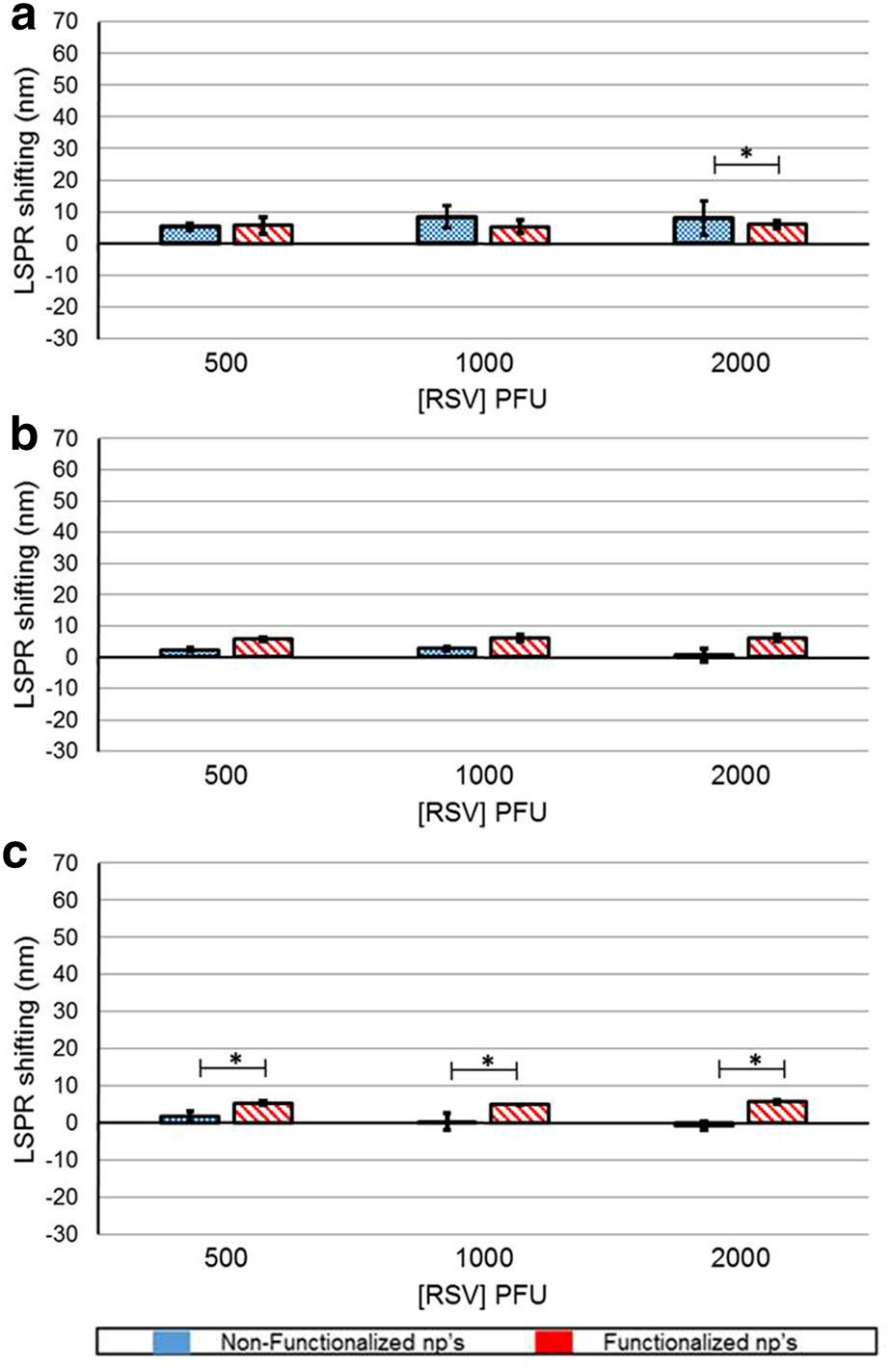
*Graph* illustrates the LSPR shifting at different titres of RSV at 30 min (**a**), 60 min (**b**) and 120 min (**c**) for antibody-functionalized (*red*) and non-functionalized (*blue*) silver nanoparticles. The *asterisk* symbol represents the significance p < 0.05

The functionalized silver nanoparticles did not show any significant LSPR shifting at 30 and 60 min time point, however at 120 min, there was significant shifting at all RSV titers. The NP did not any shifting, except an outlier for 2000 PFU RSV at 30 min (Fig. 6).

**Fig. 7:**
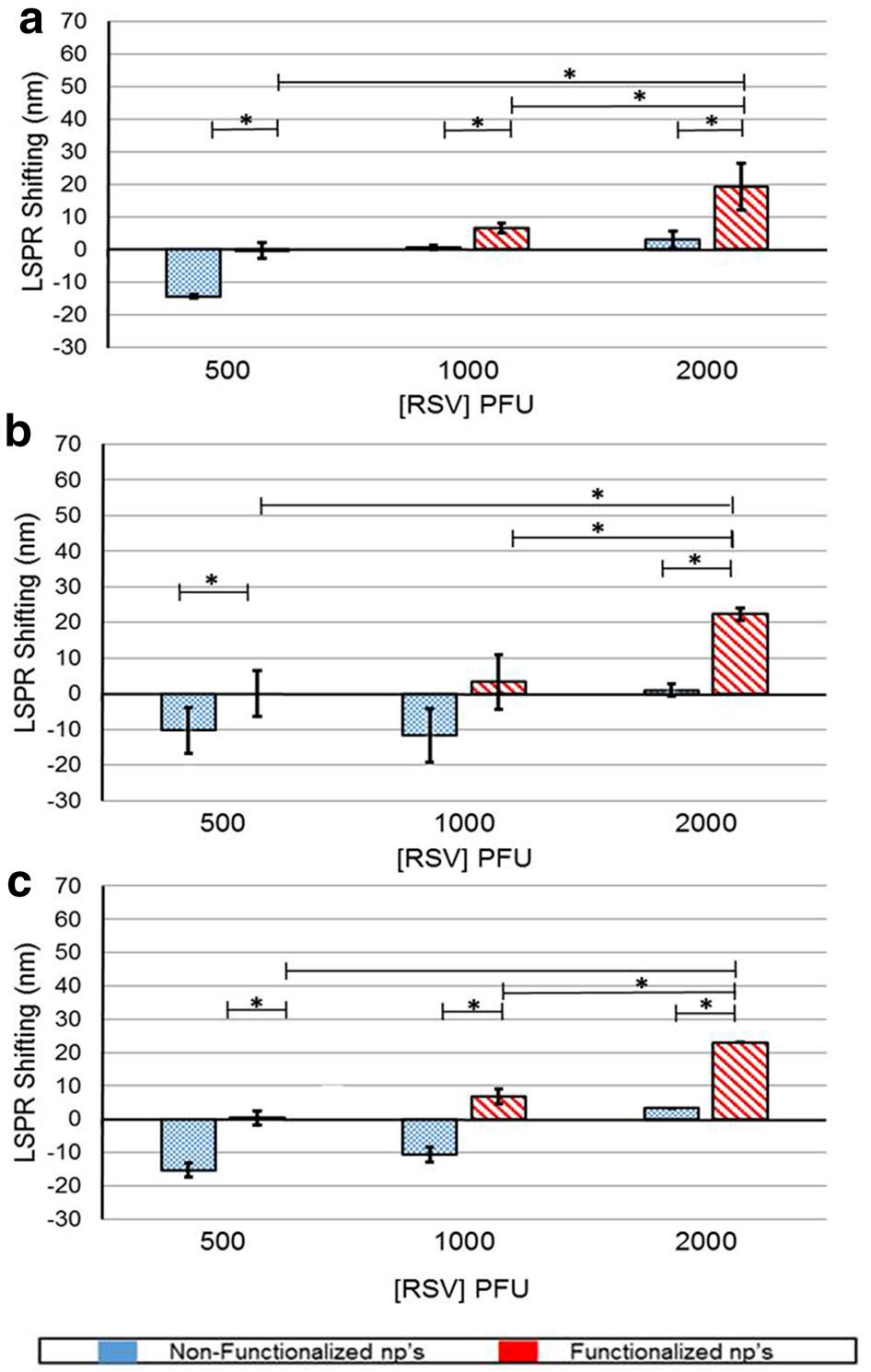
*Graph* illustrates the LSPR shifting at different titers of RSV at 30 min (**a**), 60 min (**b**) and 120 min (**c**) for antibody-functionalized (*red*) and non-functionalized (*blue*) gold nanoparticles. The *asterisk* symbol represents the significance p < 0.05

The functionalized gold nanoparticles did not show any significant LSPR shifting for 500 PFU RSV at any time point and a marginal shift for 1000 PFU RSV at all time points. There was a significant shift observed at 2000 PFU RSV and did not change with the increasing contact time (Fig. 7). At all-time points, the non-functionalized gold nanoparticles exhibited blue shifting for 500 PFU RSV; and also at 60 and 120 min, there was such blue shift for 1000 PFU RSV.

Based on these time and dose dependent study for RSV detection using fNPs, Pearson’s correlation was calculated (Table 1) and limit of detection (LOD) and limit of quantification (LOQ) values were determined (Table 2). The best possible Pearson’s co-efficient for functionalized copper and silver nanoparticles was 0.97 at 120 min and 0.87 at 60 min, respectively. However, for gold it was in the range of 0.94–0.97 at all-time points.

**Table 1:** Pearson correlation coefficient for RSV titer (PFU) and time for antibody functionalized copper, silver and gold nanoparticles

FNP	Time (min)	Pearson’s R
Cu	30	−0.76
	60	−0.79
	120	0.97
Ag	30	0.50
	60	0.87
	120	0.50
Au	30	0.97
	60	0.94
	120	0.97

**Table 2:** Limit of detection and limit of quantification values for RSV detection using antibody functionalized nanoparticles

fNP	Time (min)	LOD (PFU)	LOQ (PFU)
Cu	120	2.4	14
Ag	60	7	385
Au	120	211	640

### Specificity and cross-reactivity

Based best Pearson’s linearity for the fNPs, the specificity and cross-reactivity were investigated in presence of *P. aeruginosa* and adenovirus. The functionalized copper nanoparticles (120 min), silver nanoparticles (60 min) and gold nanoparticles (30 min) were interacted with RSV and *P. aeruginosa* or adenovirus.

**Fig. 8:**
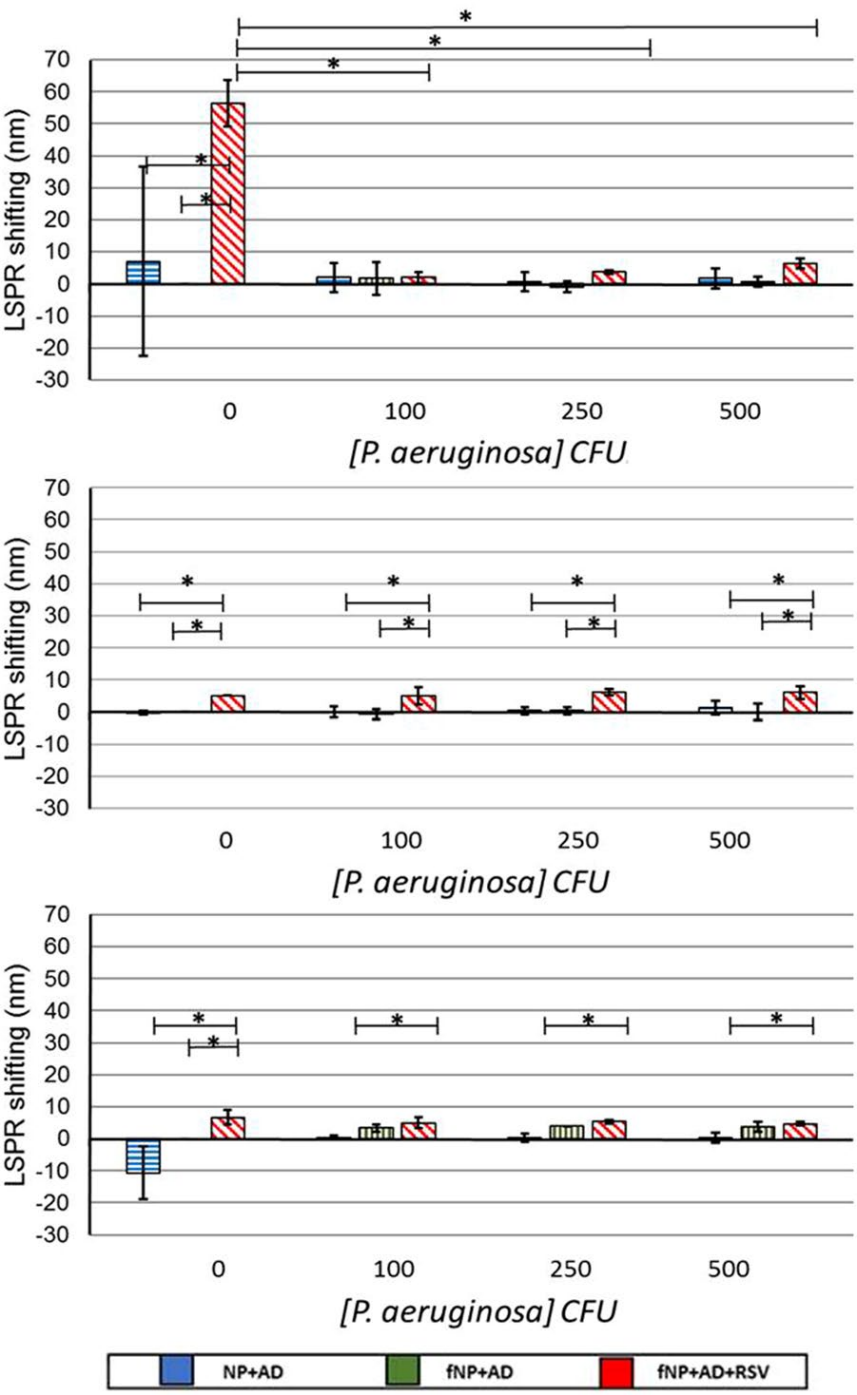
*Graph* illustrates the evaluated cross-reactivity and specificity of antibody functionalized nanoparticles in presence of *P. aeruginosa* (100, 250 and 500 CFU) against the chosen best Pearson’s linearity, using 1000 PFU of RSV for copper at 120 min (**a**), silver at 60 min (**b**) and gold nanoparticles at 30 min (**c**). The experimental setup was viz., non-functionalized nanoparticles and *P. aeruginosa* (*blue*), (ii) antibody functionalized nanoparticles and *P. aeruginosa* (*green*), (iii) antibody functionalized nanoparticles, RSV and *P. aeruginosa* (*red*). NP and fNP represent the non-functionalized and functionalized nanoparticles respectively and Bc the bacteria. The *asterisk* symbol represents the significance p < 0.05

**Fig. 9:**
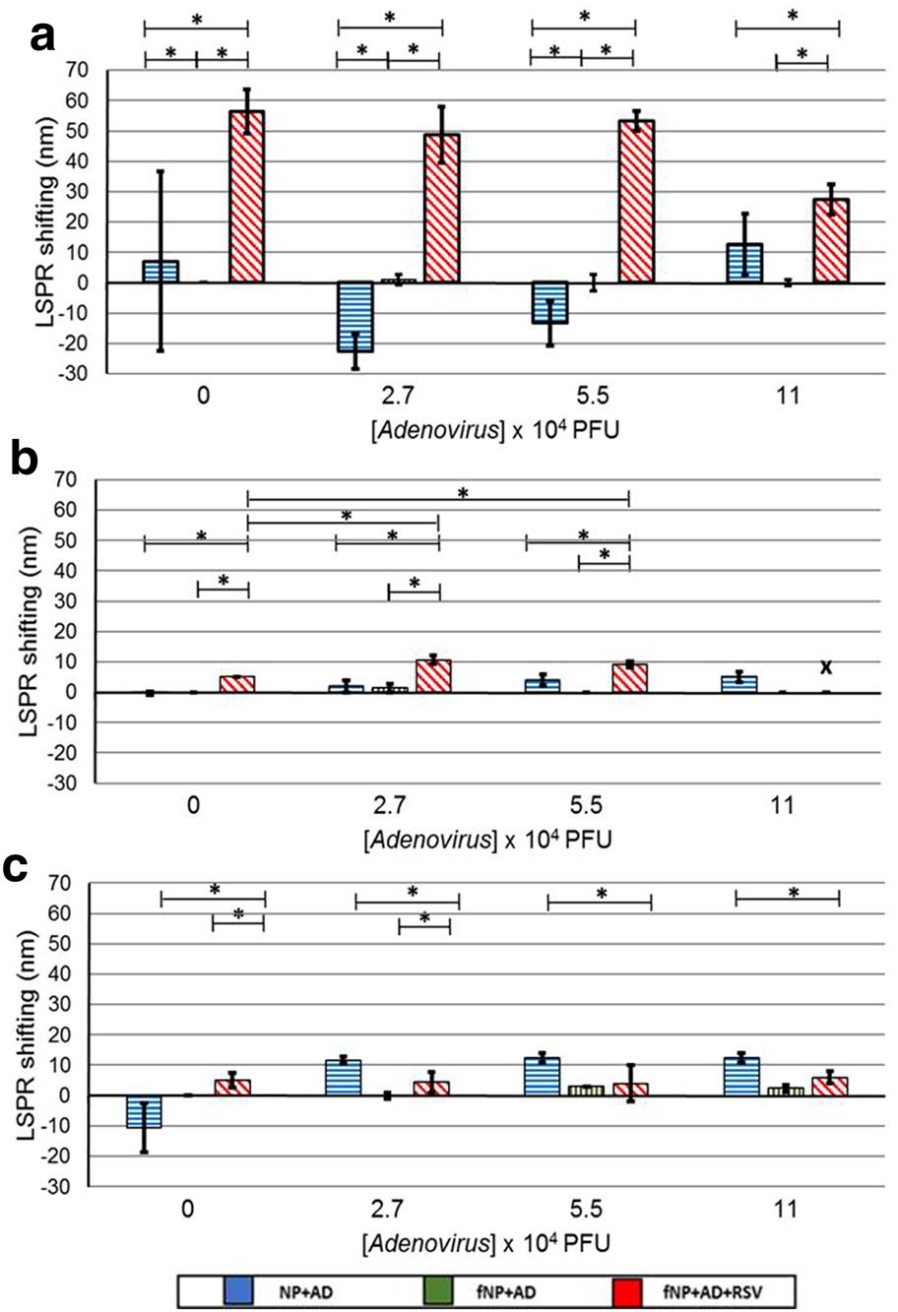
*Graph* illustrates the evaluated cross-reactivity and specificity of antibody functionalized nanoparticles in presence of Adenovirus (2.7 × 10^4^, 5.5 × 10^4^, 11 × 10^4^ PFU) against the chosen best Pearson’s linearity using 1000 PFU of RSV for **a** copper at 120 min, **b** silver at 60 min and **c** gold nanoparticles at 30 min with adenovirus. The experimental setup was viz., (i) non-functionalized nanoparticles and adenovirus, (ii) antibody functionalized nanoparticles and adenovirus, (iii) antibody functionalized nanoparticles, RSV and adenovirus. NP and fNP represent the non-functionalized and functionalized experiments respectively and AD for Adenovirus. The *asterisk* symbol represents the significance p < 0.05

The non-functionalized and functionalized copper nanoparticle did not show any shift in presence of *P. aeruginosa* or RSV and *P. aeruginosa* together (Fig. 8a). However, there was no cross-reactivity for adenovirus and the fNPs specifically detected RSV in the presence of adenovirus (Fig. 9a). In case of silver nanoparticles, the fNP showed specificity for RSV in presence of *P. aeruginosa* (Fig. 8b) and adenovirus (Fig. 9b) by exhibiting significant LSPR shifting with no cross-reactivity. The non-functionalized and functionalized gold nanoparticles showed marginal shifting in for *P. aeruginosa* and adenovirus, suggesting cross-reactivity and lack of specificity towards RSV (Figs. 8c, 9c).

**Fig. 10:**
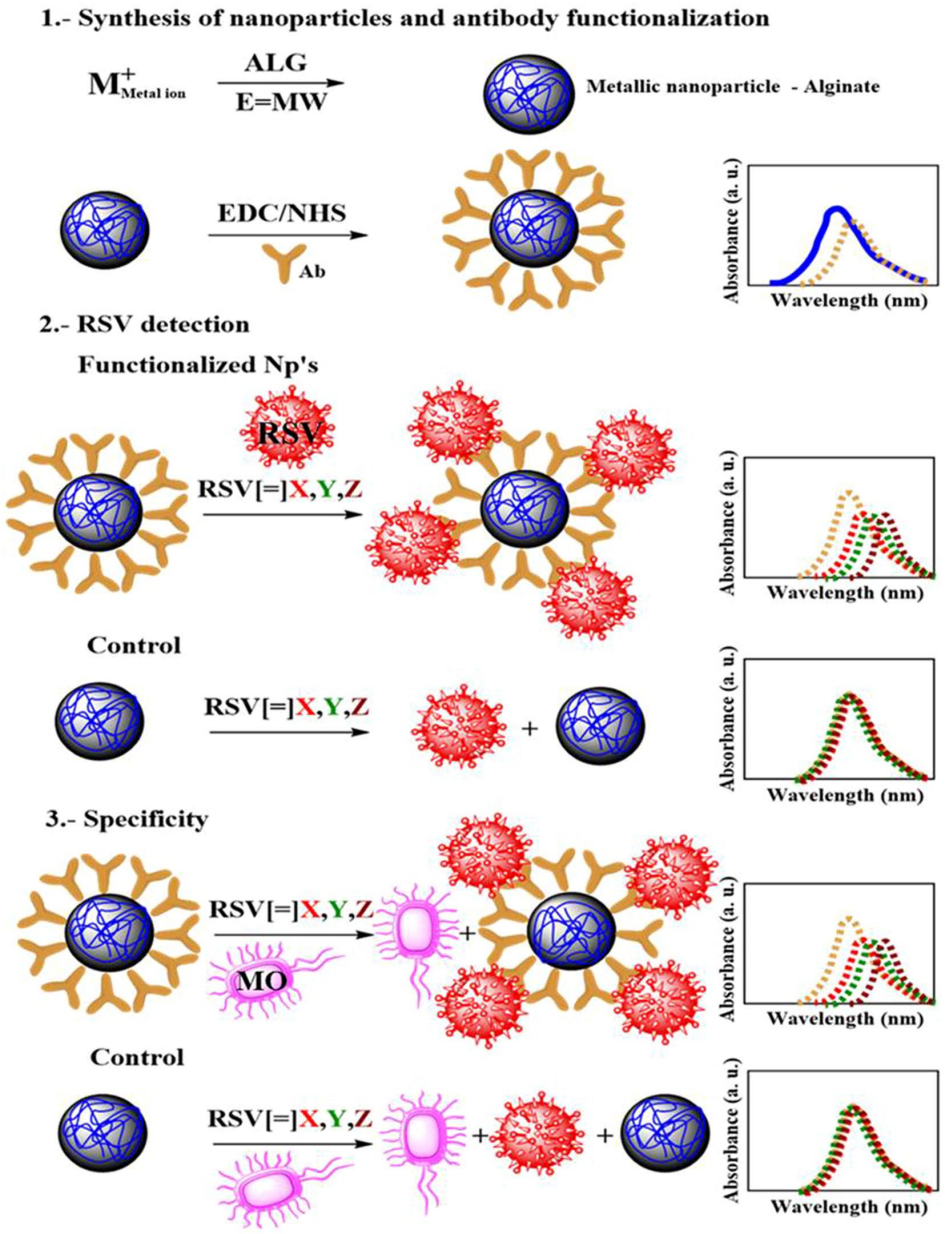
Schematic overview of current study showing the LSPR behaviour of metallic nanoparticles upon surface modification with antibody. The interactions of functionalized nanoparticles with RSV and under the influence of microbial organism (MO: *Pseudomonas aeruginosa* and adenovirus) related to the shifting of UV-Vis spectra

All the UV-visible analyses of the fNP and NP and their interaction with the RSV detection and specificity are summarized in the Fig. 10. The UV-visible spectra for these experiments are provided as Additional file 2 and Additional file 3 for *P. aeruginosa* and adenovirus, respectively.

## Discussion

The biophysical methods for RSV detection are mainly PCR-ESI-MS and SERS, there is need for an alternative method to expand the ambit of RSV detection regime. The applications of metallic nanoparticles are extensive in the field of sensing and nanomaterial based sensors are widely used for development of cost-effective detection devices. SERS and LSPR are the popular sensing methods, which exploit the physicochemical peculiarity of particular metallic nanomaterials. The SERS utilizes metallic nanomaterial for detection of various analytes specifically even at extremely low levels, however, it is not cost-effective. The advantage of SERS can be offered by a simpler technique of LSPR spectroscopy [40]. LSPR based detection methods have been used for detection of hepatitis B virus [41], human immunodeficiency virus [26] and influenza virus [3, 42]. However, this study reports the LSPR based RSV detection using anti-RSV antibody functionalized metallic nanoparticles.

Generally, functionalization of the nanoparticle increases their size and consequently the width of the LSPR. Therefore, it is important to determine an optimal functionalization that provides scope for measurable LSPR (Fig. 1). The nature of nanoparticles and amount of functionalized biomolecules determine the LSPR, however the confounder may be non-specific interactions between nanoparticles may lead to agglomeration and affect the LSPR pattern (Fig. 1a) [43]. Our alginate reduced and stabilized copper, silver and gold nanoparticles size corroborate with the previous reports [44–46], as the specific plasmonic absorption relates to the nano-metric size (Fig. 1). However, the particle size distribution indicates agglomeration of NP and fNP (Fig. 2) and as expected, the functionalization of nanoparticles reduced the zeta potential of the nanoparticles (Fig. 3). This may be the result of non-specific interactions (H-bonds, carboxyl groups, cross-linking) of alginate or antibody functionalization [47, 48]. We performed FE-SEM analyses to confirm the size of nanoparticles and it was found to be smaller than 100 nm, however some agglomeration was evident (Fig. 4).

Time and titer dependent RSV detection with copper, silver and gold nanoparticles showed varied results. The interaction of RSV and antibody functionalized copper showed highest shifting as compared to silver and gold. The shifting was in the range of 45–60 nm at all time points (Fig. 5). At 120 min, the functionalized copper nanoparticles showed highest linear correlation with the RSV titer (Table 1) with a LOD and LOQ of 2.4 and 14, respectively (Table 2). The functionalized silver nanoparticles did not interact prolifically with RSV as with the copper (Fig. 6). However, there was a shifting specific for RSV and at 60 min, the LOD and LOQ values were calculated as 7 and 385 respectively (Tables 1, 2). The gold nanoparticles functionalized with antibody resulted in shifting of LSPR in response to RSV binding. The shifting was more than silver nanoparticles; however, this system was better at higher RSV titer and more contact time (Fig. 7). Although, it has a convincing linearity, the LOD and LOQ values are 211 and 640, respectively (Tables 1, 2). The differences in these values of RSV shifting by copper, silver and gold nanoparticles (fNPs) can be due to the peculiar LSPR profile and the amount antibody functionalized on the nanoparticles.

For development of effective detection method, it is important to evaluate the cross-reactivity and specificity of the system. Therefore, we assessed fNP against respiratory pathogen *P. aeruginosa* and adenovirus for cross-reactivity and RSV specificity. We found that the fNP did not cross-react with the *P. aeruginosa* (Fig. 8) and adenovirus significantly (Fig. 9). However, the non-functionalized gold nanoparticles interacted marginally with *P. aeruginosa* and adenovirus as a result of non-specific interactions between the alginate coated nanoparticles and *P. aeruginosa* mucoid cell wall and adenovirus.

The specificity of our nanoparticles system to detect RSV in presence of *P. aeruginosa* and adenovirus is one of the highlights of the study. Functionalized copper and silver nanoparticles were able to specifically detect RSV even in the presence of *P. aeruginosa* and adenovirus, however the gold nanoparticles showed non-specific shifting (Figs. 8, 9). It should be noted that the size of but *P. aeruginosa* is (1.5–3.0 µm × 0.5–0.8 µm), almost 10 times bigger than RSV (150–300 nm). The titer of *P. aeruginosa* (1 − 5 × 10^2^ CFU) and Adenovirus (2.7 × 10^4^ – 11 × 10^4^ PFU) for the specificity experiments is considerably large. These factors adversely impact the LSPR shifting. However, we could still detect RSV and it shows the potential of LSPR detection system.

Overall, our study shows that metallic nanoparticles could be fabricated specifically for RSV detection; however, it is important to consider factors like suitable material, size, shape, contact time and LSPR behavior of nanoparticles for customized applications.

## Conclusions

There is need for development of easy and rapid detection devices for respiratory pathogens like RSV, considering the fact that cost-effective and early detection of etiological agent results in effective treatment. Our results demonstrated the efficacy of antibody functionalized metallic (copper, silver and gold) nanoparticles for detected RSV using a simple and easy procedure of UV-visible spectroscopy. Detection of RSV in the presence of non-specific entities shows the potential of the LSPR based detection systems. Thus, LSPR is an easy and rapid alternative method for development of new detection devices for RSV.

## Methods

### Synthesis of metallic nanoparticles

The synthesis of metallic nanoparticles (NP) was carried out following a modified methodology from Kalwar et al. [49]. Briefly, in a beaker, 9 mL of ethylene glycol, 0.6 mL of sodium alginate (10 mM) and 0.3 mL of NaCO_3_ were added (0.1 M), mixed and the pH was adjusted at 11, 10 and 12.5 for copper, silver and gold nanoparticles synthesis, respectively. To this, 1 mL of 10 mM of CuSO_4_·5H_2_O, 1.8 mg of AgNO_3_ and 1 mL of HAuCl_4_ was added to respective beakers for copper, silver and gold nanoparticles synthesis. Then, the beakers were placed in the microwave (MW) for 3, 1 and 1 min for copper, silver and gold, which results the solutions to change the color to reddish, grey and purple for each system, respectively. At that point, the solutions were centrifuged at 10,000 rpm for 30 min for copper and silver and for gold NPs were centrifuged at 8000 rpm for 30 min. The nanoparticles were then washed with distilled water 3 times using the same corresponding centrifuge conditions as mentioned above. Pelleted nanoparticles were suspended in distilled water (for copper and gold 0.5 mL water and 2 mL for silver).

### Functionalization of nanoparticles with antibody

The functionalization was done using 1-Ethyl-3-(3-dimethylaminopropyl) carbodiimide (EDC) chemistry. Filter sterilized 200 µL of EDC (1 mg/ml), 200 µL of nanoparticles (16.9 ± 0.39, 15.7 ± 0.17 and 8.3 ± 0.3 mg) were placed in microcentrifuge tubes, and then 2.5, 5 and 10 µL of polyclonal antibody (4 mg/mL) was added. The different amount of antibody was used to optimize the LSPR for each nanoparticle system. The solution was vortexed for 10 s and then placed on a titer plate shaker at room temperature for 3 h. Microcentrifuge tubes were centrifuged at 8000 rpm for 30 min and the supernatant was removed and stored (to be used for protein estimation). This process was repeated two more times and the pellet (nanoparticles) was re-suspended in 200 µL of distilled water.

Non-functionalized (NP) and functionalized nanoparticles (fNP) were sonication for 10 s and analyzed by UV-visible spectroscopy (Beckman Coulter DU 800 Spectrophotometer), by taking 10 µL of nanoparticles and making the volume to 50 µL using distilled water.

### Particle size distribution and zeta potential

The particle size distribution and zeta potential of non-functionalized and fNP were measured using a Zetasizer (Nano-ZS; Malvern Instruments Ltd, Malvern, UK). The nanoparticle solutions were diluted in distilled water, placing 50 µL of the sample in 2 mL of distilled water (pH of 6.58 ± 0.23). The measurements for each sample were repeated three times.

### Determination of antibody functionalized on the nanoparticles

The BCA protocol was used to quantify the antibody attached to the nanoparticles following manufacturer’s instructions (Thermo Fisher Scientific, NY, USA). Briefly, 150 µL of each standard (bovine serum albumin) and samples (washes from the fNPs) were placed into a microplate. Then, 150 µL of the working reagent was added to each well and mixed thoroughly for 30 s. The microplate was covered and incubated at 37 °C for 2 h. Then, the absorbance was measured at 562 nm on a plate reader.

### FE-SEM analysis

For FE-SEM analysis, an aluminum substrate previously polished and washed with distilled water and acetone for three times was used. A drop of the nanoparticle’s solution was placed on the substrate and it was allowed to evaporate at room temperature and imaged using JEOL JSM-7401f microscope (JEOL USA, Inc. MA, USA).

### RSV, Adenovirus and *Pseudomonas aeruginosa*

Human respiratory syncytial virus (ATCC^®^ VR-26™), human adenovirus 5 ATCC^®^ VR-5™ and *P. aeruginosa* (Schroeter) Migula (ATCC^®^ 39324™) a mucoid strain were procured from American Type Culture Collection (ATCC) and maintained as instructed by ATCC. RSV and adenovirus were obtained in eagle’s minimum essential medium supplemented with 2 % FBS. While, *P. aeruginosa* was grown overnight in LB broth, pelleted, washed and suspended in sterile distilled water.

### LSPR based RSV detection

The detection of RSV was performed using 10 µL of LSPR optimized fNPs (5 µL of Ab for copper and gold and 10 µL for silver) to which 2.5 µL (500 PFU), 5 µL (1000 PFU) and 10 µL (2000 PFU) of RSV was added and incubated at room temperature on a shaker for 30, 60 and 120 min. Distilled water was then adding to tubes to make up the volume of 60 µL and analyzed using a UV-vis spectrophotometer. Similarly, 10 µL of NPs were also interacted with RSV as control.

### Evaluating the specificity and cross-reactivity of fNPs

In order to assess the specificity of RSV detection for copper, silver and gold fNPs, the LSPR shifting was measured under the influence of *P. aeruginosa* and *Adenovirus*. The cross-reactivity of fNP was tested by observing the shifting upon *P. aeruginosa* and *Adenovirus* addition. Therefore, the experimental setup was (i) NPs + *P. aeruginosa*, (ii) fNPs + *P. aeruginosa*, (iii) fNPs + RSV + *P. aeruginosa*. Similar experimental setup was designed for Adenovirus. The dose of *P. aeruginosa* used for this experiments was 1 µL (100 CFU), 2.5 µL (250 CFU) or 5 µL (500 CFU); and 2.5 µL (2.7 × 10^4^ PFU), 5 µL (5.5 × 10^4^ PFU) or 10 µL (1.1 × 10^5^ PFU) of the Adenovirus. Based on the best linearity (Pearson’s R) of the corresponding nanoparticles (fNPs) the time and titer (RSV) were selected.

The LSPR was measure using UV-visible spectroscopy as described earlier in the RSV detection section. Briefly, in a microcentrifuge tube, 10 µL of the NPs/fNPs and *P. aeruginosa* were allowed to interact and also in the other tube, fNPs were allowed to incubate with RSV and *P. aeruginosa* together. Finally, the volume was increased to 60 µL and absorbance was measured. In the same manner, specificity and cross-reactivity of fNPs in presence of adenovirus was evaluated.

### Statistical analysis

All the experiments were performed in triplicates and the results were analyzed using OriginLab™ 9 software and presented as mean ± standard deviation. Results were subjected to two-way ANOVA, and a Tukey test was applied for RSV detection and specificity experiments. The differences were significant at p < 0.05 (*).

The limit of detection and quantification were calculated for the experiments at different times which had the best linearity (120 min for copper, 60 min for silver and 30 min for gold) using the following equation [50]:

1. LOD = 3.3 (σ_(x, y)_/slope)
2. LOQ = 10 (σ _(x, y)_/slope)

## Additional files

10.1186/s12951-016-0167-z UV-vis spectra for the RSV detection.
10.1186/s12951-016-0167-z UV-vis spectra for the specificity and cross-reactivity experiments for *P. aeruginosa.*

10.1186/s12951-016-0167-z UV-vis spectra for the specificity and cross-reactivity experiments for adenovirus.

## Abbreviations

[RSV]: RSV titer
RSV: respiratory syncytial virus
Ab: antibody
BCA: bicinchoninic acid
CFU: colony forming units
DFA: direct florescent antibody
DNA: deoxyribonucleic acid
EDC: 1-Ethyl-3-(3-dimethylaminopropyl) carbodiimide
ELISA: enzyme-linked immunosorbent Assay
HIV: human immunodeficiency virus
LOD: limit of detection
LOQ: limit of quantification
LSPR: localized superficial plasmon resonance
MW: microwave
fNP: functionalized nanoparticles
NP: non-functionalized nanoparticle
PCR: polymerase chain reaction
PFU: plaque forming units
QD: quantum dots
SERS: surface-enhanced Raman spectroscopy
FESEM: field-emission scanning electron microscopy
UV-vis: ultraviolet-visible
