# Supplementary material for: Facile and rapid detection of respiratory syncytial virus using metallic nanoparticles
Source: J Nanobiotechnology. 2016 Feb 27;14:13. doi: 10.1186/s12951-016-0167-z (PMC4769566; doi:10.1186/s12951-016-0167-z)
Supplement: Supplementary file 1 — 10.1186/s12951-016-0167-z UV-vis spectra for the RSV detection. [file 12951_2016_167_MOESM1_ESM.pdf]

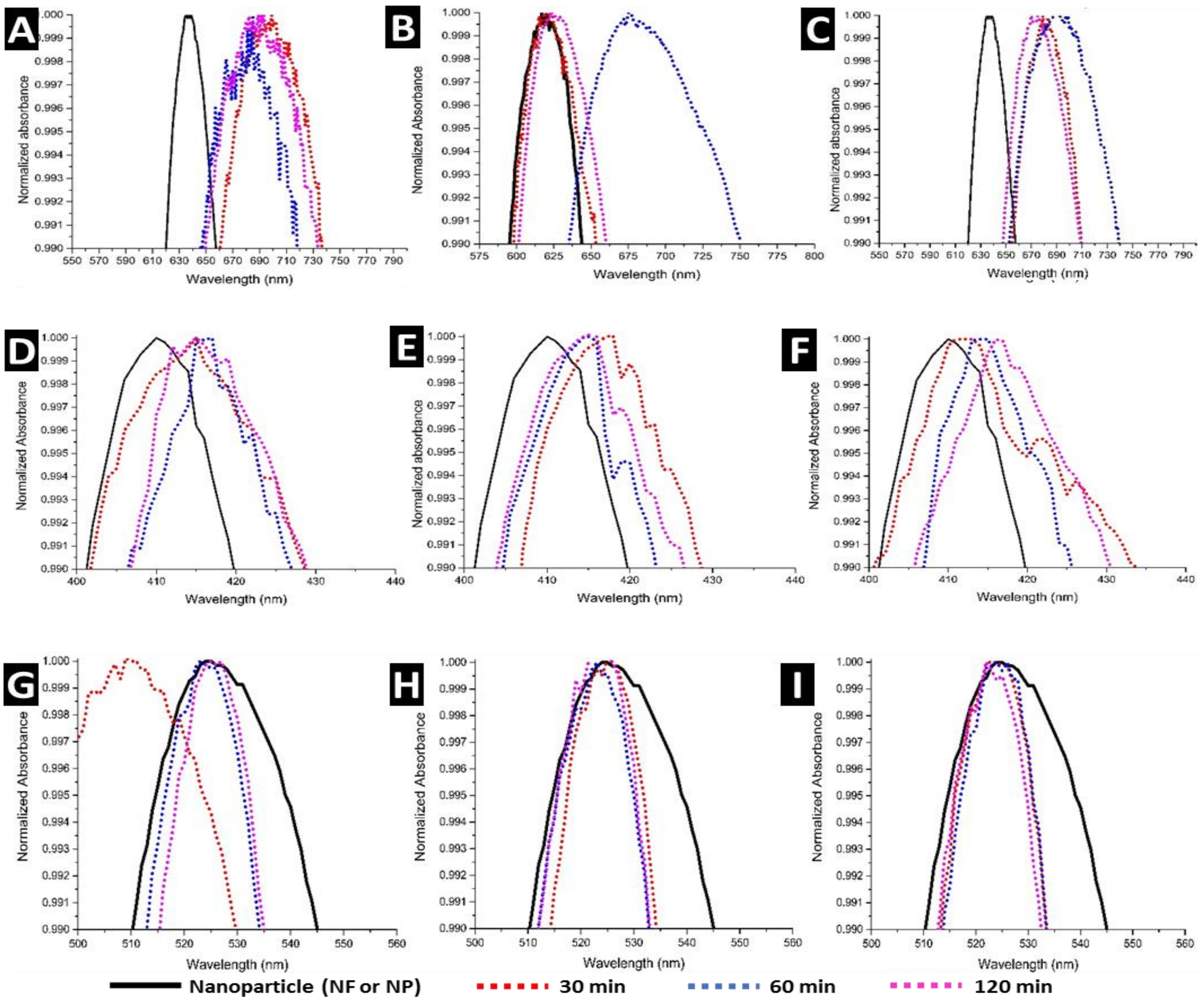

UV-vis analysis for the detection of 500 PFU of RSV at 30, 60 and 120 min. using functionalized copper (A, B and C) silver (D, E and F) and gold nanoparticles (G, H and I) with 3 replicates.

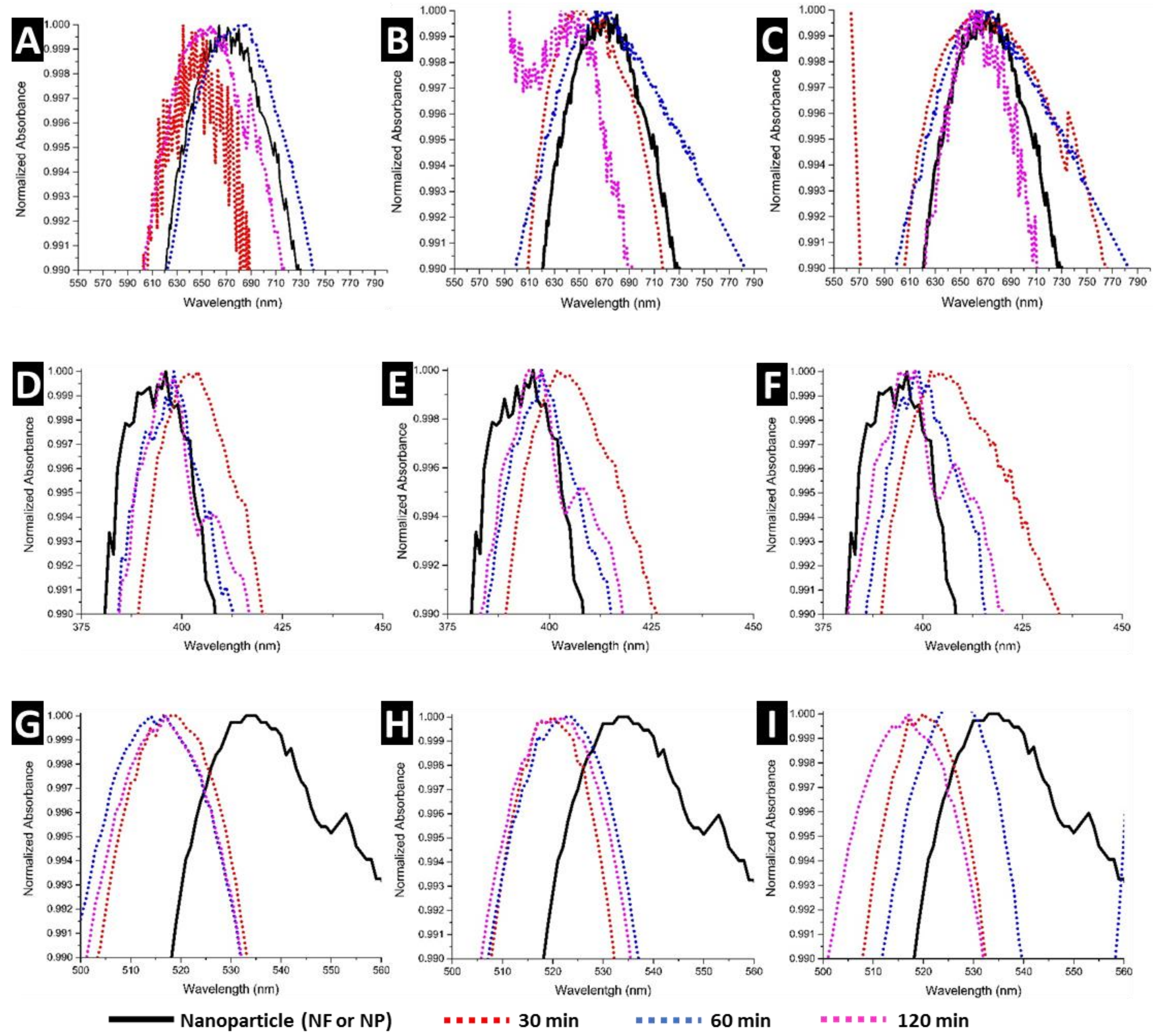

UV-vis analysis for the detection of 500 PFU of RSV at 30, 60 and 120 min. using copper (A, B and C) silver (D, E and F) and gold nanoparticles (G, H and I) with 3 repetitions.

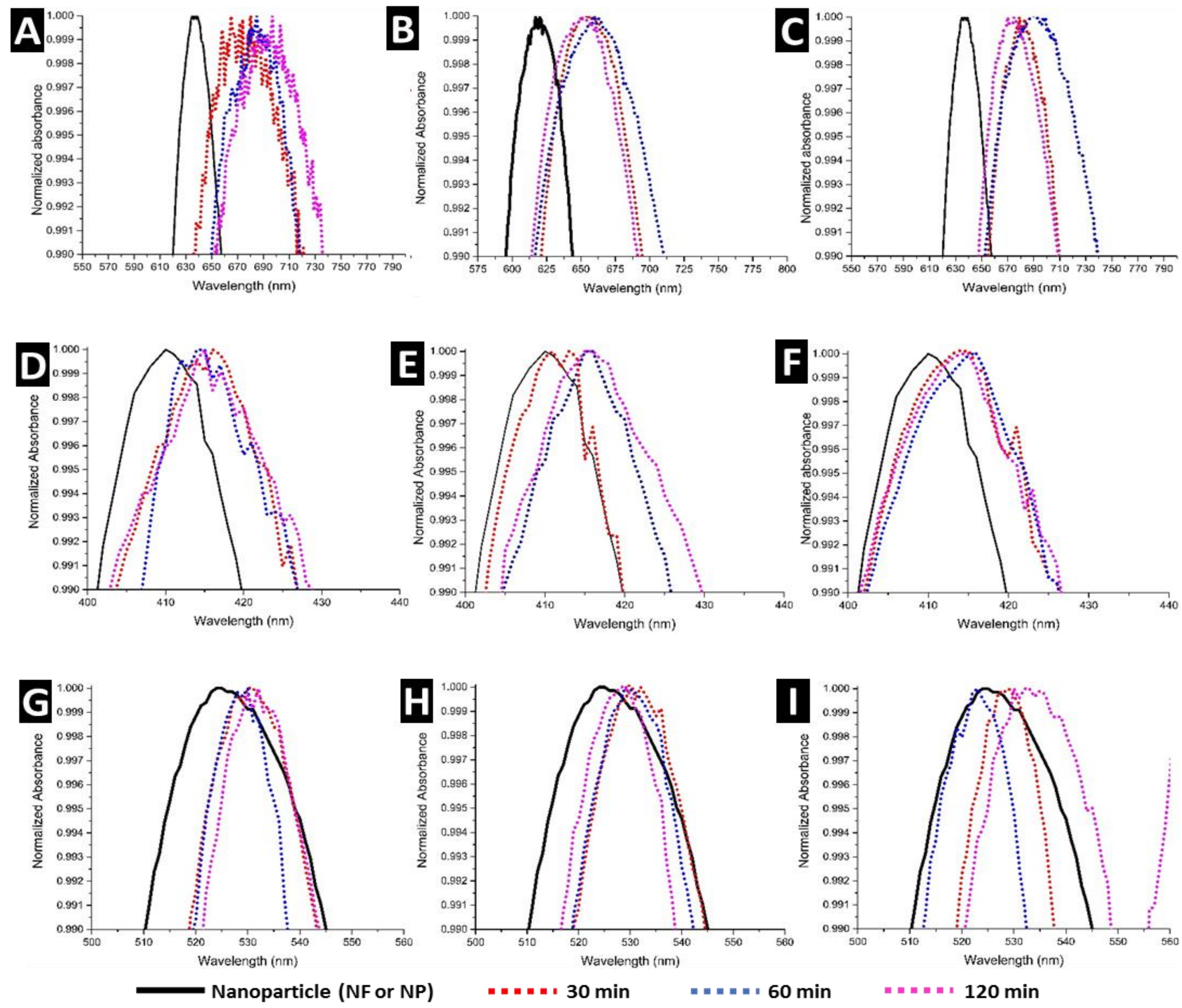

UV-vis analysis for the detection of 1000 PFU of RSV at 30, 60 and 120 min. using functionalized copper (A, B and C) silver (D, E and F) and gold nanoparticles (G, H and I) with 3 repetitions.

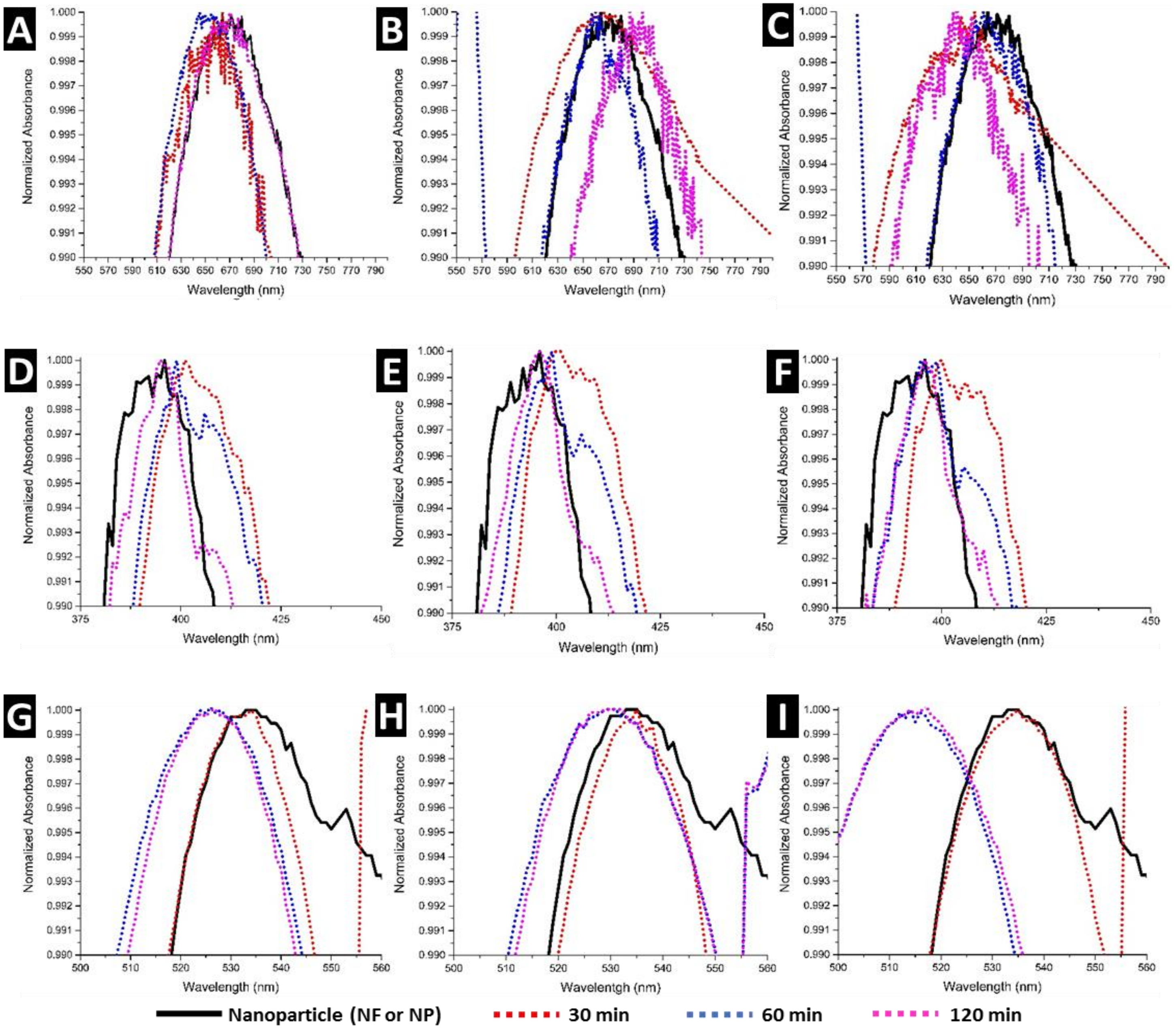

UV-vis analysis for the detection of 1000 PFU of RSV at 30, 60 and 120 min using copper (A, B and C) silver (D, E and F) and gold nanoparticles (G, H and I) with 3 repetitions.

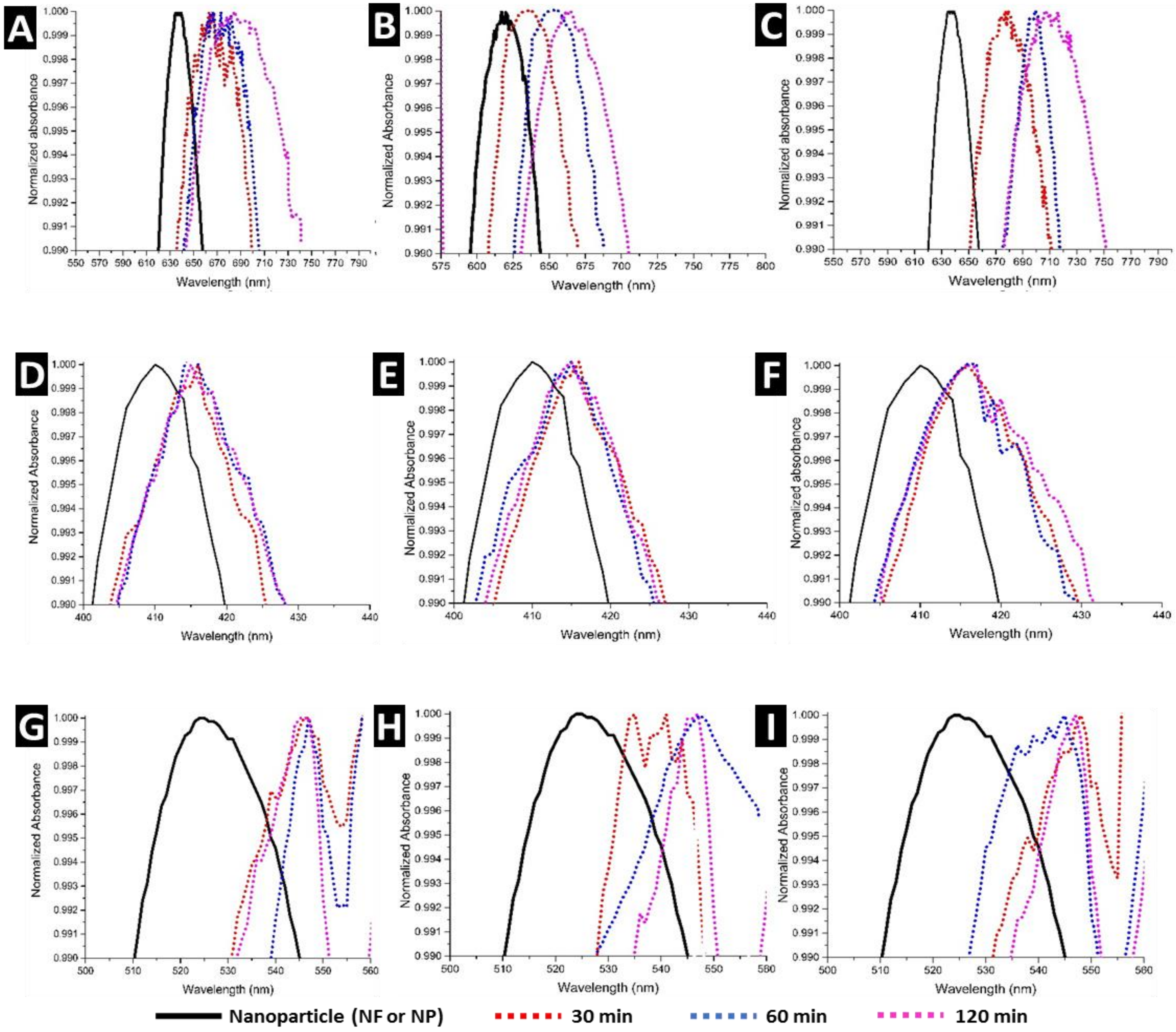

UV-vis analysis for the detection of 2000 PFU of RSV at 30, 60 and 120 min. using functionalized copper (A, B and C) silver (D, E and F) and gold nanoparticles (G, H and I) with 3 repetitions.

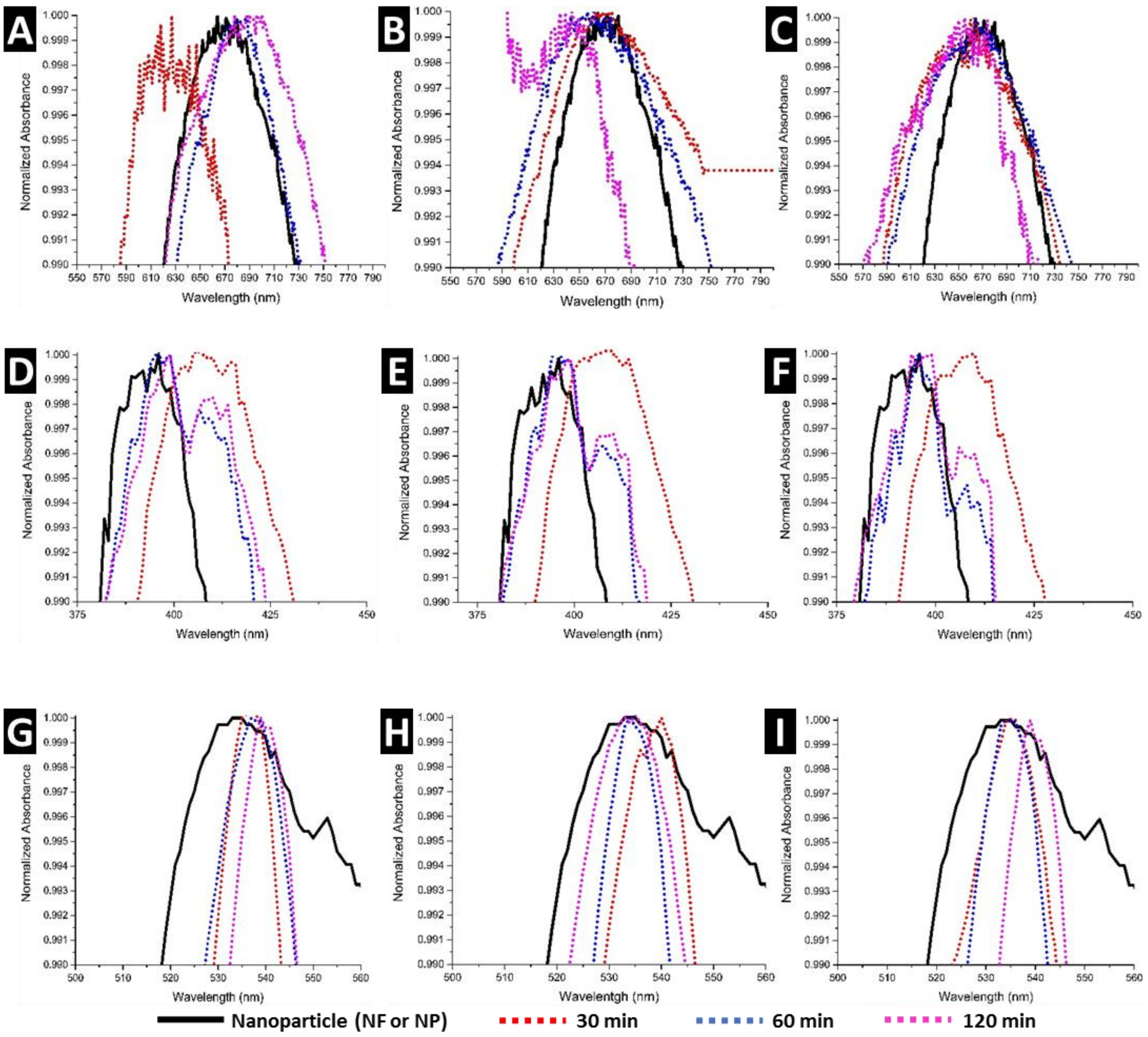

UV-vis analysis for the detection of 2000 PFU of RSV at 30, 60 and 120 min. using copper (A, B and C) silver (D, E and F) and gold nanoparticles (G, H and I) with 3 repetitions.
