# Supplementary material for: Facile and rapid detection of respiratory syncytial virus using metallic nanoparticles
Source: J Nanobiotechnology. 2016 Feb 27;14:13. doi: 10.1186/s12951-016-0167-z (PMC4769566; doi:10.1186/s12951-016-0167-z)
Supplement: Supplementary file 2 — 10.1186/s12951-016-0167-z UV-vis spectra for the specificity and cross-reactivity experiments for P. aeruginosa. [file 12951_2016_167_MOESM2_ESM.pdf]

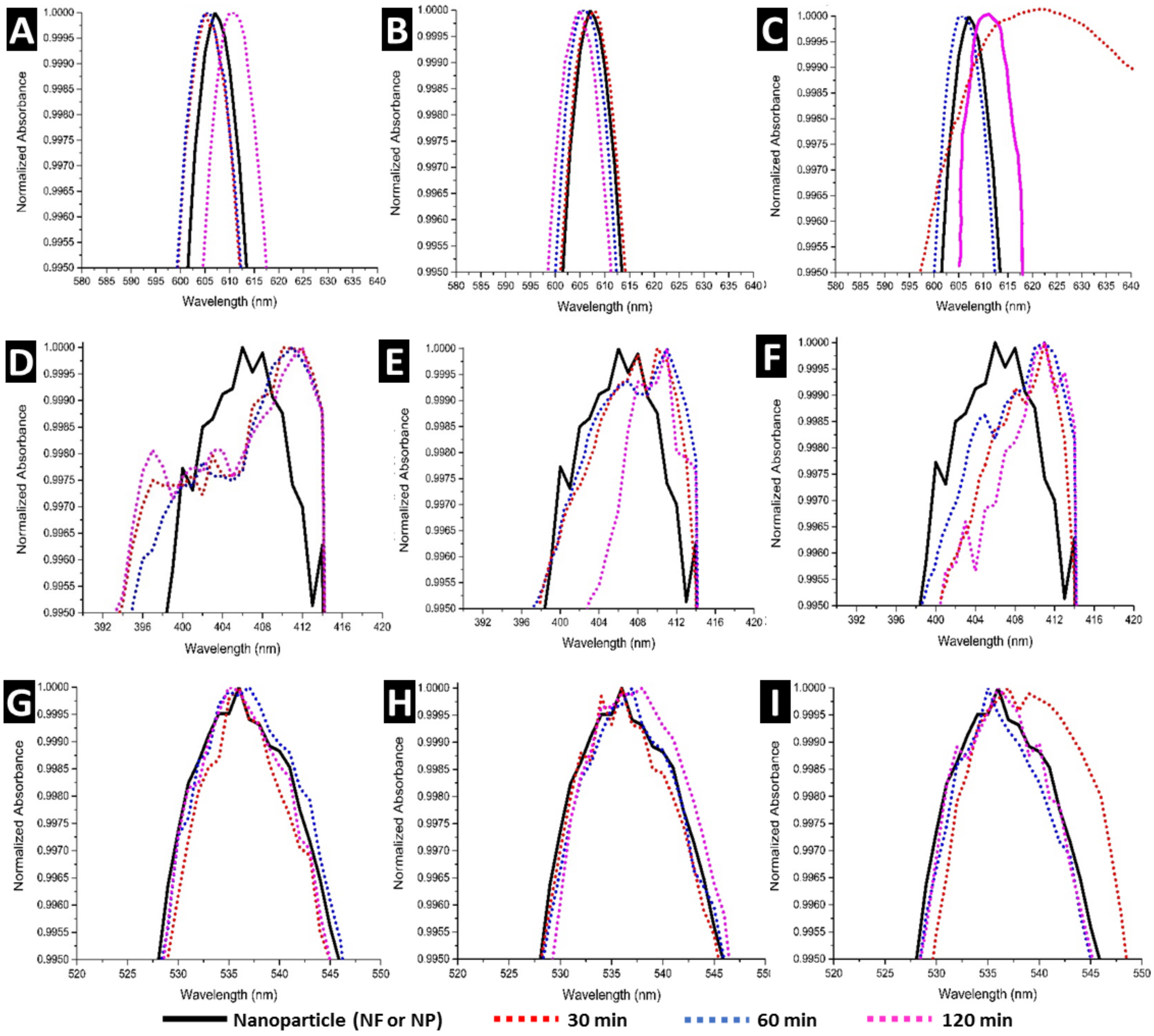

Figure 21 UV-vis analysis to evaluate cross-reactivity of non-functionalized copper (A, B and C) silver (D, E and F) and gold nanoparticles (G, H and I) towards *P. aeruginosa* (3 replicates).

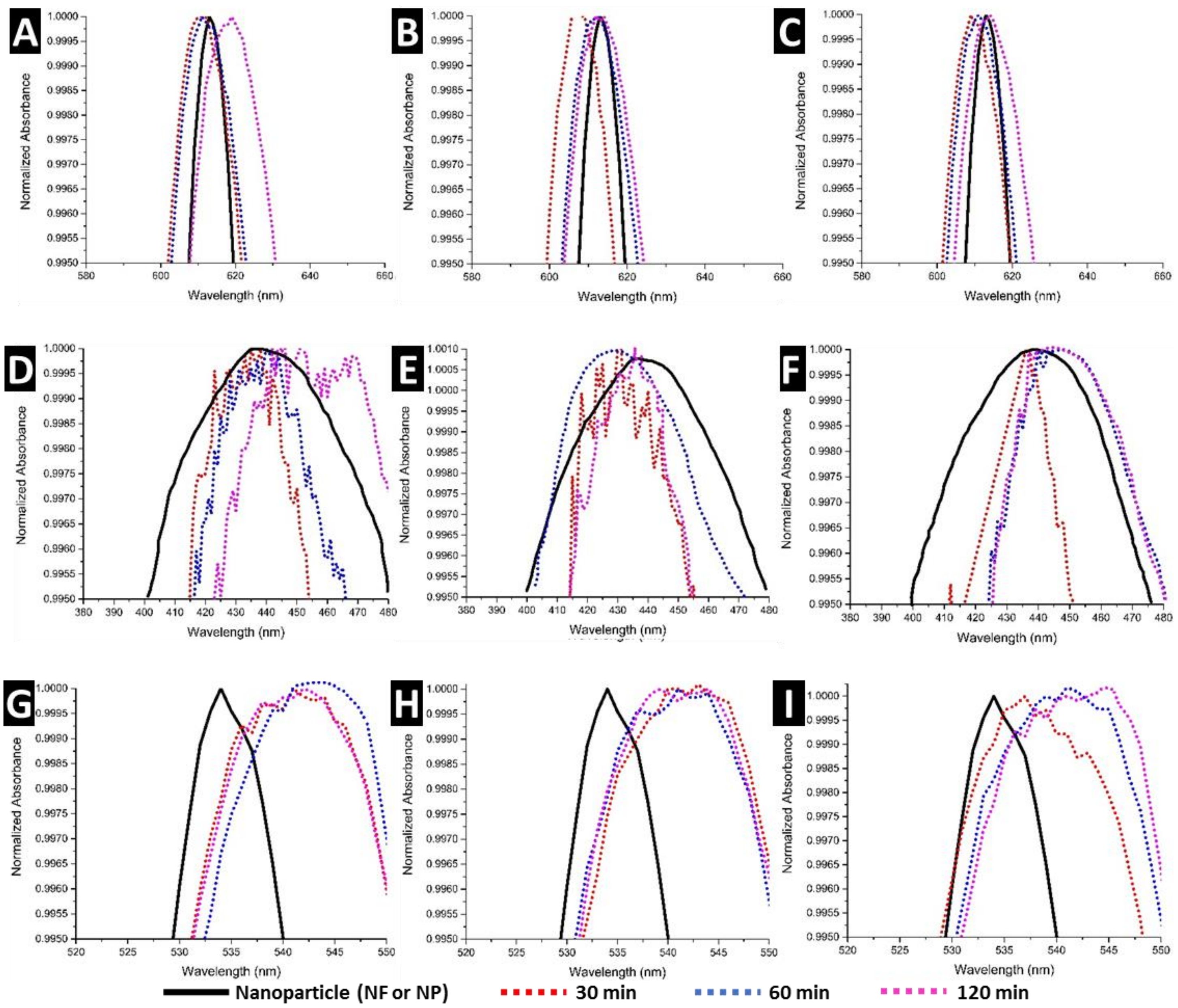

Figure 22 UV-vis analysis to evaluate cross-reactivity of functionalized copper (A, B and C) silver (D, E and F) and gold nanoparticles (G, H and I) towards *P. aeruginosa* (3 replicates).

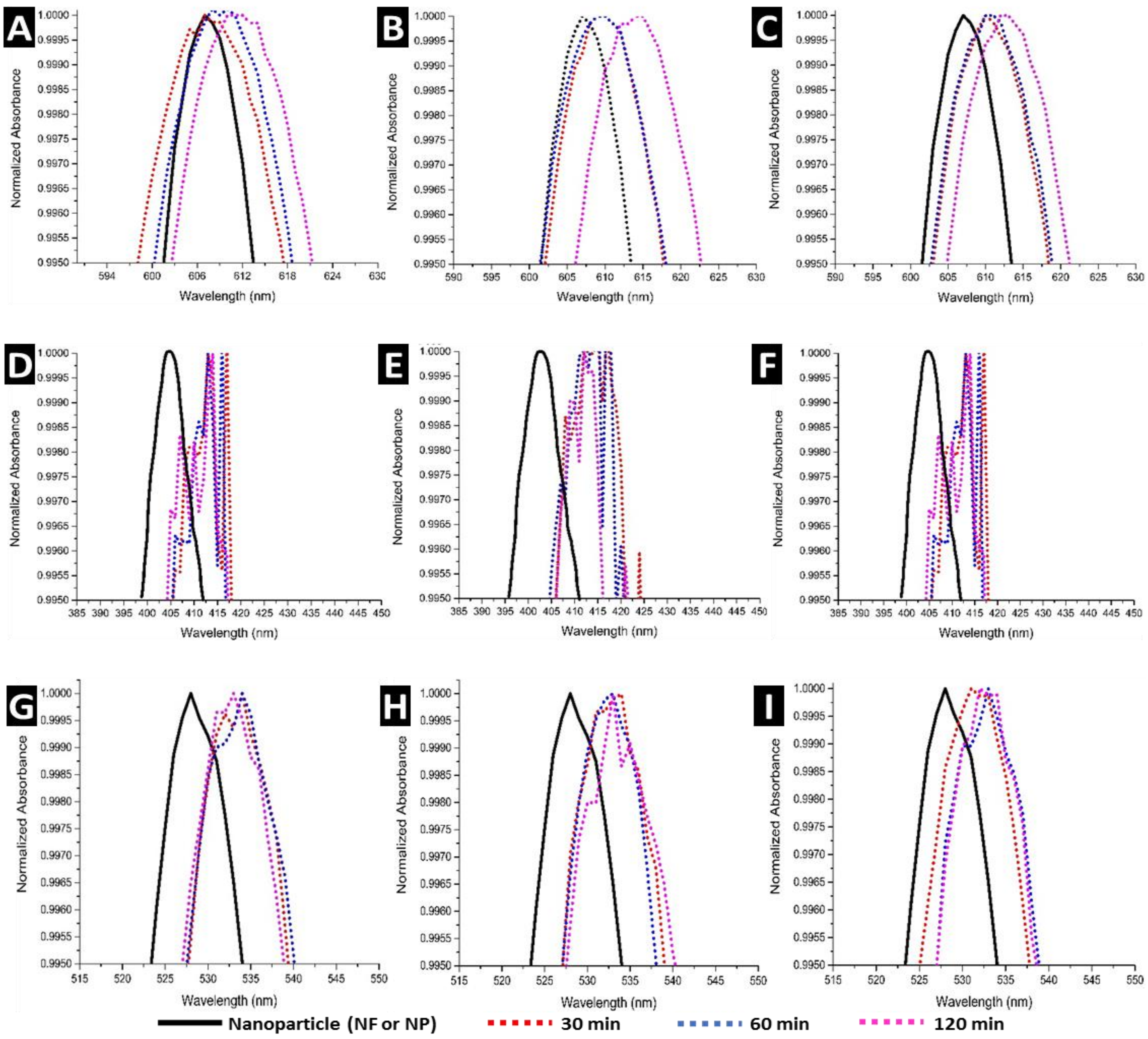

Figure 23 UV-vis analysis to evaluate specificity towards RSV by the functionalized copper (A, B and C) silver (D, E and F) and gold nanoparticles (G, H and I) in presence of *P. aeruginosa* (3 replicates).
