# Supplementary material for: Facile and rapid detection of respiratory syncytial virus using metallic nanoparticles
Source: J Nanobiotechnology. 2016 Feb 27;14:13. doi: 10.1186/s12951-016-0167-z (PMC4769566; doi:10.1186/s12951-016-0167-z)
Supplement: Supplementary file 3 — 10.1186/s12951-016-0167-z UV-vis spectra for the specificity and cross-reactivity experiments for adenovirus. [file 12951_2016_167_MOESM3_ESM.pdf]

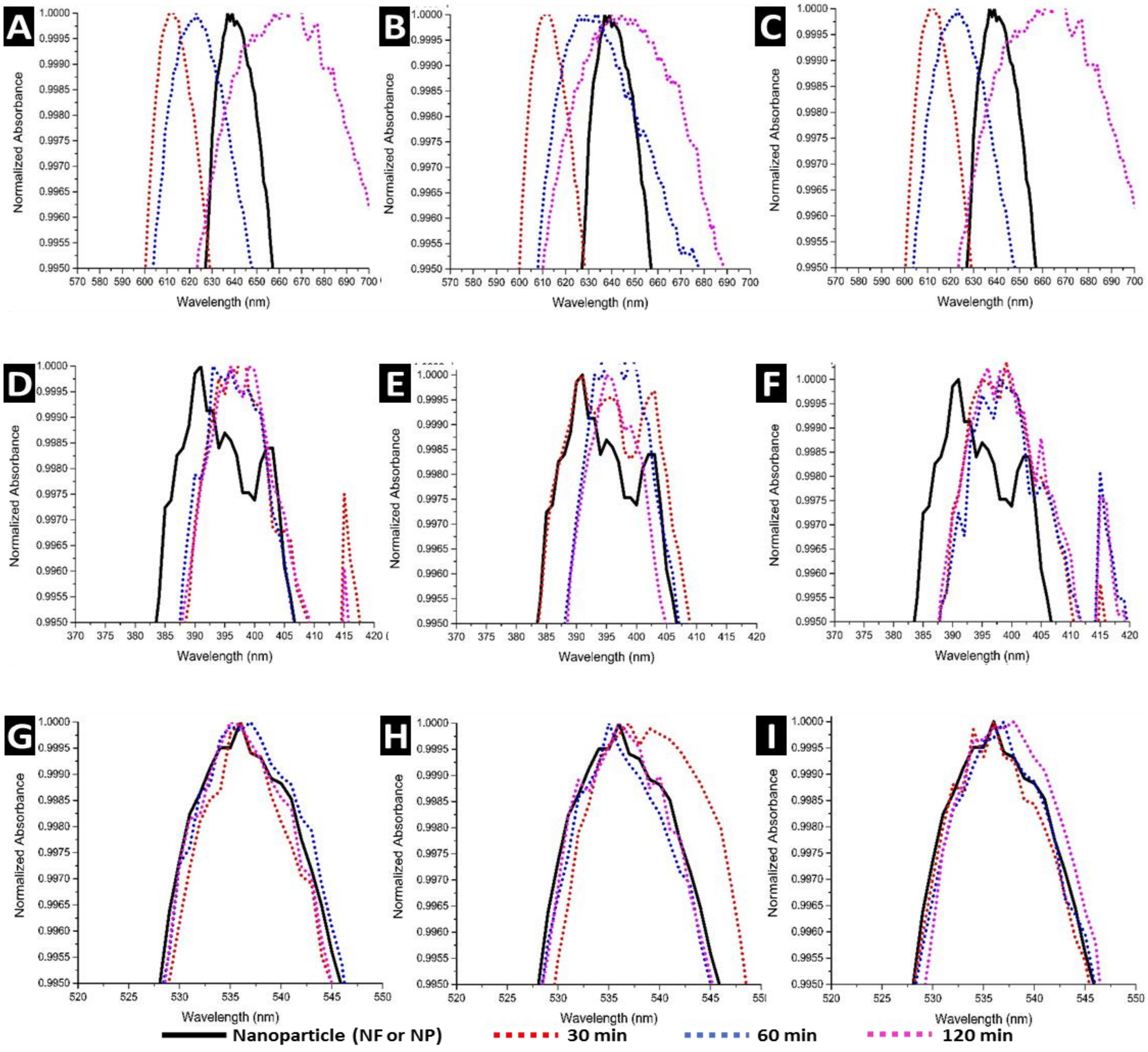

UV-vis analysis to evaluate cross-reactivity of non-functionalized copper (A, B and C) silver (D, E and F) and gold nanoparticles (G, H and I) towards Adenovirus (3 replicates).

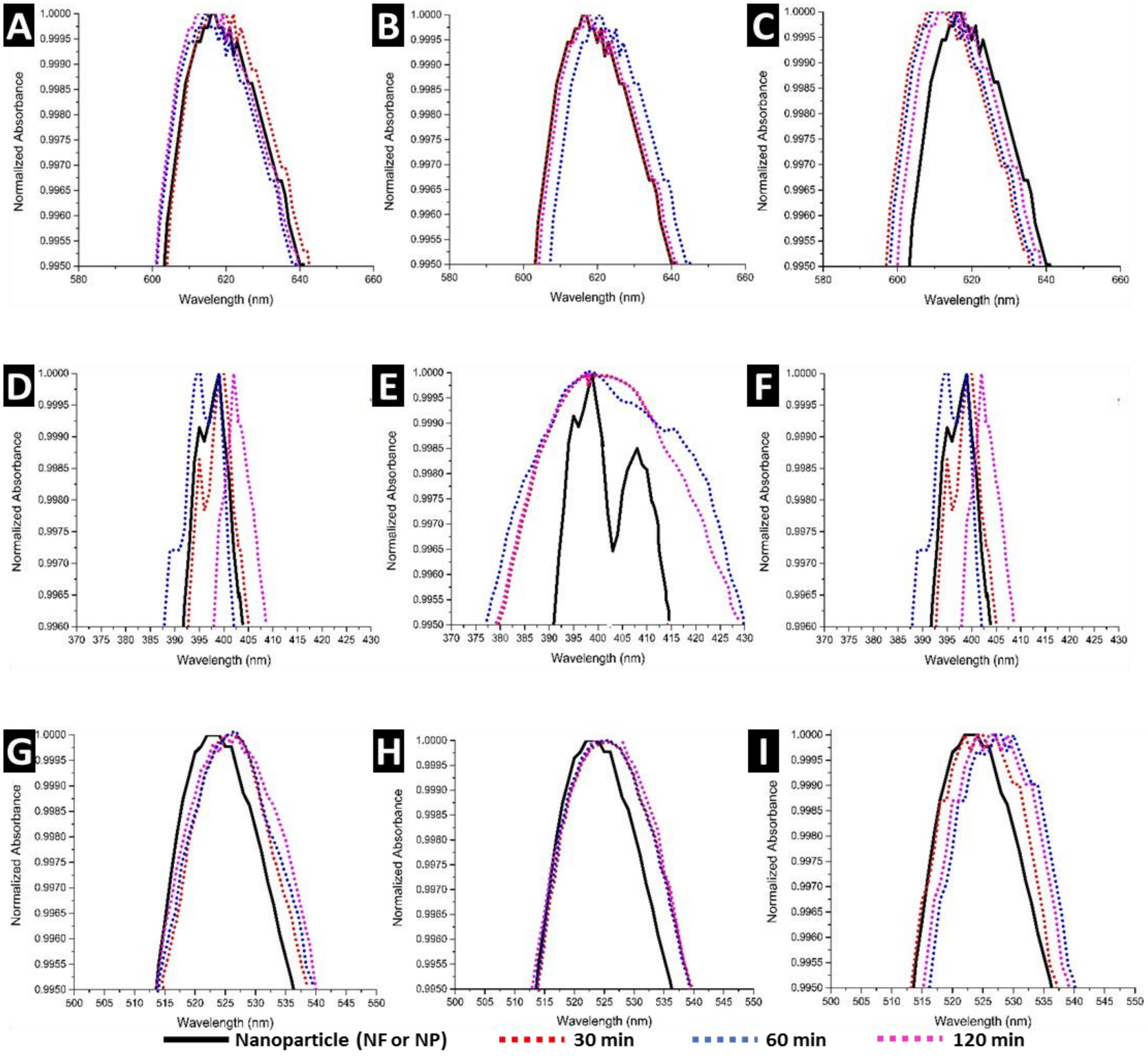

UV-vis analysis to evaluate cross-reactivity of functionalized copper (A, B and C) silver (D, E and F) and gold nanoparticles (G, H and I) towards Adenovirus (3 replicates).

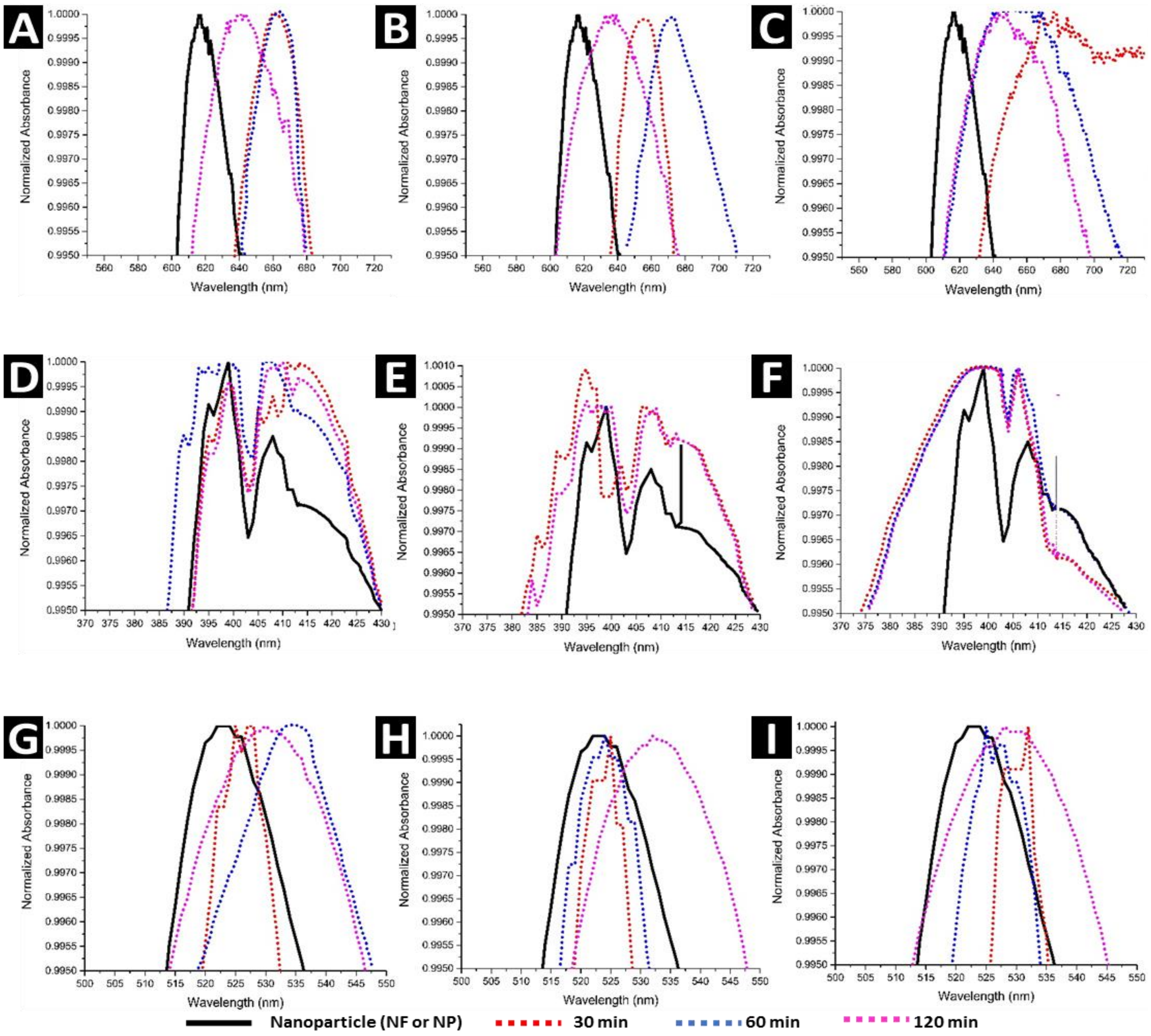

UV-vis analysis to evaluate specificity towards RSV by the functionalized copper (A, B and C) silver (D, E and F) and gold nanoparticles (G, H and I) in presence of Adenovirus (3 replicates).
